# Changpu Yujin Decoction Alleviates Tourette Syndrome by Modulating Lipid Metabolism and Microglial Polarization: Involvement of the TREM2/NF-κB Signaling Axis

**DOI:** 10.3390/ph19101529

**Published:** 2026-09-26

**Authors:** Jingxi Yao, Yushu Wei, Fu Zhang, Wenjun Yuan, Yu Li, Kexin Sun, Xing Wei, Shuang Huang, Jialin Zhong, Jing Shang, Zhenggang Shi

**Affiliations:** 1 School of Clinical Chinese Medicine, Gansu University of Chinese Medicine, 35 Dingxi East Road, Chengguan District, Lanzhou 730000, China; yaojx2pdcm@163.com (J.Y.); weiyushu_tcm@163.com (Y.W.); zyzf369@163.com (F.Z.); eidolon830126@163.com (W.Y.); 13893560267@163.com (Y.L.); 13895089534@163.com (K.S.); weixing0219@163.com (X.W.); hscool164@163.com (S.H.); 2 School of Clinical Medicine, Chengdu University of Traditional Chinese Medicine, 37 Shi’erqiao Road, Jinniu District, Chengdu 610075, China; jialinzhong2022@163.com

**Keywords:** Changpu Yujin Decoction, Tourette syndrome, lipid metabolism, microglia, TREM2/NF-κB signaling axis

## Abstract

**Background:** Changpu Yujin Decoction (CPYJD) has been clinically utilized for more than ten years as an effective herbal formula for Tourette syndrome (TS). However, the exact mechanism by which CPYJD treats TS via modulating lipid metabolism and microglia (MG) polarization remains unclear. **Methods:** A combination of in vivo and in vitro experiments was employed. A total of 72 three-week-old Sprague–Dawley (SD) rats were divided into a control group (*n* = 12) and a modeling group (*n* = 60). After successful model establishment, the modeled rats were randomly divided into five groups (*n* = 12 per group): the model group, the tiapride group, and the low-dose, medium-dose, and high-dose Changpu Yujin Decoction (CPYJD-L, CPYJD-M, and CPYJD-H, respectively) groups. All groups were administered the corresponding treatments for 28 consecutive days, and therapeutic efficacy was evaluated by behavioral scoring after the intervention. Neuronal morphology and myelin structure were observed by hematoxylin–eosin (HE) staining, Nissl staining, and transmission electron microscopy (TEM). The expression of lipid metabolism- and microglial phenotype-related indicators in each group was detected by immunofluorescence, immunohistochemistry, enzyme-linked immunosorbent assay (ELISA), biochemical assays, reverse transcription quantitative PCR (RT-qPCR), and Western blotting (WB). In addition, lipidomics analysis was performed to identify differential lipid metabolites. For the in vitro experiments, triggering receptor expressed on myeloid cells 2 (TREM2) was knocked down in BV2 cells using small interfering RNA (siRNA), and inflammation was induced by lipopolysaccharide (LPS). The cells were divided into a control group, an LPS group, a CPYJD-medicated serum group, a TREM2 knockdown group, and a TREM2 knockdown plus CPYJD-medicated serum group. Cell morphology was observed under a microscope, and lipid metabolism- and microglial phenotype-related indicators in each group were detected by immunofluorescence, biochemical assays, RT-qPCR, and WB. **Results:** Compared with the control group, rats in the model group exhibited significantly increased locomotor activity and stereotyped behaviors, injured striatal neurons with myelin damage, abnormal lipid metabolism and lipid accumulation, an enhanced M1-like pro-inflammatory phenotype with elevated inflammatory cytokine levels, downregulated TREM2 expression, and activation of the nuclear factor kappa-B (NF-κB) signaling pathway. CPYJD reduced stereotyped behaviors and locomotor activity in TS model rats, alleviated neuronal and myelin injury, improved lipid metabolic abnormalities and lipid accumulation, enhanced the M2-like anti-inflammatory phenotype, reduced inflammatory cytokine levels, and upregulated TREM2 expression while inhibiting the activation of the NF-κB signaling pathway. TREM2 knockdown aggravated lipid metabolic abnormalities, lipid accumulation, and the expression of M1-like pro-inflammatory phenotype-related factors in BV2 cells, whereas CPYJD-medicated serum reversed these changes. **Conclusions:** CPYJD ameliorated pathological injury of striatal neurons, improved lipid metabolic abnormalities and lipid accumulation in MG, promoted a shift in MG from the M1-like pro-inflammatory phenotype toward the M2-like anti-inflammatory phenotype, and reduced inflammatory cytokine levels, thereby alleviating tic symptoms in TS model rats. These effects may be associated with the involvement of the TREM2/NF-κB signaling axis.

## 1. Introduction

Tourette syndrome (TS) is a prevalent neurodevelopmental disorder (NDD) that typically onsets in childhood and adolescence. Its core clinical features are defined by involuntary, abrupt, rapid, and recurrent motor tics accompanied by vocal tics in affected children [1]. Epidemiological studies show that the global prevalence of TS is approximately 0.5%, with a pronounced gender disparity: males exhibit a significantly higher disease prevalence than females [2]. As TS shares susceptibility genes with other NDDs, it frequently presents with comorbid neurodevelopmental conditions, including attention-deficit/hyperactivity disorder (ADHD) and obsessive-compulsive disorder (OCD) [3,4,5], which severely impair children’s physical and mental health as well as social functioning. Nevertheless, the pathogenesis of TS remains incompletely elucidated. The current consensus attributes TS primarily to dysfunctional dopaminergic neurotransmission within the cortico-striatal-thalamic-cortical (CSTC) circuit [6,7]. The contributions of immune, inflammatory, and genetic factors to TS development have also been increasingly validated [8,9,10].

Recent studies have suggested that lipid metabolic dysregulation and neuroinflammation may be involved in the pathological process of TS. Neuroimaging studies have revealed widespread structural abnormalities in the cortico-striato-pallido-thalamic white matter pathways of patients with TS [11]. Abnormalities in lipid metabolism and organic acid metabolism have also been observed in the 3,3’-iminodipropionitrile (IDPN)-induced TS mouse model [12]. Single-cell transcriptomic and epigenomic analyses of TS patients have shown that microglia (MG) are in an activated state, with upregulated expression of immune system-related genes [13]. Microglial activation and increased neuroinflammation have also been observed in the brains of IDPN-induced TS model rats [14]. Myelin is a multilayered lamellar membrane structure that wraps around neuronal axons in the nervous system; it not only maintains axonal structural integrity but also participates in the regulation of higher cognitive functions [15]. MG, the resident macrophages of the central nervous system (CNS), play an important role in remyelination by clearing myelin debris from demyelinating lesions [16,17]. Triggering receptor expressed on myeloid cells 2 (TREM2) is a transmembrane protein predominantly expressed on the microglial surface; it is involved in the regulation of cholesterol, myelin, and phospholipid metabolism and drives the transition of MG toward a disease-associated microglia (DAM) phenotype [18]. Aberrant TREM2 expression can lead to microglial lipid metabolic dysregulation and phenotypic alterations, and further activate the downstream nuclear factor kappa-B (NF-κB) pathway, a classic inflammatory signaling pathway [19,20]. Studies have demonstrated that abnormal TREM2 expression is involved in the pathogenesis of multiple NDDs [21,22].

Current treatments for TS are dominated by behavioral interventions and pharmacotherapy. Behavioral interventions, such as habit reversal training (HRT) and Comprehensive Behavioral Intervention for Tics (CBIT), are effective for mild-to-moderate tics with favorable safety profiles; however, a considerable proportion of patients fail to achieve complete symptom relief following behavioral therapy. Moreover, behavioral therapy primarily reduces tic severity and hardly ameliorates the common comorbidities and core internalizing problems of TS (such as emotional dysregulation), and access is limited by the scarcity of trained therapists [23]. Regarding pharmacological treatments, dopamine receptor antagonists (such as aripiprazole and tiapride) are currently recommended as first-line agents, while α_2_-adrenergic receptor agonists (such as clonidine and guanfacine) are particularly suitable for adolescent patients with comorbid ADHD. Although these agents effectively reduce tic severity, dopamine receptor blockers are frequently associated with adverse effects including metabolic disturbances, weight gain, sedation, and extrapyramidal symptoms, and noradrenergic agents may also cause drowsiness and hypotension [7], leading to self-discontinuation and poor long-term adherence. Therefore, developing alternative therapies with definite efficacy and improved safety remains an urgent need in the clinical management of TS. Traditional Chinese medicine (TCM) confers unique advantages in TS management. It has been demonstrated to significantly reduce Yale Global Tic Severity Scale (YGTSS) scores in TS patients, with a relatively short treatment course and milder adverse effects [24]. The American Academy of Neurology has rated the level of evidence for proprietary Chinese medicines such as Ningdong Granule and Wuling Granule in TS treatment as moderate [25].

The formula used in this study, Changpu Yujin Decoction (CPYJD), was modified by Professor Zhenggang Shi from the classical prescription recorded in *Wenbing Quanshu* (Complete Compendium of Warm Diseases), based on the traditional Chinese medicine understanding of the etiology and pathogenesis of TS as well as the physiological and pathological characteristics of children. With over 10 years of clinical application, CPYJD has shown remarkable therapeutic efficacy against TS with no notable adverse reactions [26]. Our previous in vivo studies demonstrated that the therapeutic effect of CPYJD on TS is attributed to its improvement of myelin injury and regulation of amino acid neurotransmitter disturbances in the brains of model rats, as well as the activation of PTEN-induced kinase 1 (PINK1)/Parkin pathway-mediated mitophagy, which reduces reactive oxygen species (ROS) accumulation, thereby inhibiting NOD-, LRR- and pyrin domain-containing protein 3 (NLRP3) inflammasome activation and alleviating pyroptosis [27,28]. Accordingly, we hypothesized that CPYJD may attenuate neuroinflammation by improving lipid metabolism in MG and promoting their shift from the M1-like pro-inflammatory phenotype toward the M2-like anti-inflammatory phenotype, thereby exerting an anti-tic effect, and that this effect may be associated with the TREM2/NF-κB signaling axis. The present study tested this hypothesis through in vivo and in vitro experiments.

## 2. Results

### 2.1. UPLC-MS/MS Analysis of CPYJD

The components of CPYJD were identified using ultra-performance liquid chromatography–tandem mass spectrometry (UPLC-MS/MS) in positive (Figure 1A) and negative (Figure 1B) ion modes, yielding a total of 925 annotated compounds. The relative abundance of each compound in CPYJD was determined based on peak areas, and the top 20 predominant compounds—mainly terpenoids, ketones, and glycosides—are presented in Table 1.

### 2.2. CPYJD Improves Behavioral Scores and Neuronal Pathological Manifestations in TS Rats

After intraperitoneal injection of IDPN, the motor behavior scores and stereotyped behavior scores of rats in each modeling group were significantly increased compared with those in the control group (*p* < 0.01), and all scores were ≥2, indicating successful model establishment. After 28 days of intervention, the motor behavior scores and stereotyped behavior scores of the model group remained significantly higher than those of the control group (*p* < 0.01). Compared with the model group, all treatment groups showed significantly lower behavioral scores (*p* < 0.01). Compared with the high-dose CPYJD (CPYJD-H) group, the low- and medium-dose CPYJD (CPYJD-L/M) groups showed higher behavioral scores (*p* < 0.01), whereas no significant difference was observed between the tiapride group and the CPYJD-H group (*p* > 0.05). These results indicate that both tiapride and CPYJD effectively alleviated tic symptoms in TS model rats, with CPYJD exerting its beneficial effects in a dose-dependent manner (Figure 2A,B).

Hematoxylin–eosin (HE) and Nissl staining revealed abundant striatal neurons with normal morphology and orderly, compact arrangement in the control group; Nissl bodies in the cytoplasm were abundant, uniformly stained, and clearly granulated, with regular nuclear membranes and distinct nucleoli, and no obvious nuclear pyknosis or displacement was observed. Compared with the control group, striatal neurons in the model group were sparsely and disorderly arranged, with nuclear pyknosis, cytoplasmic swelling, and neuronal degeneration and necrosis; Nissl bodies in the cytoplasm were reduced in number, lightly stained, or even dissolved and disappeared, and the Nissl-positive area was significantly decreased (*p* < 0.01). After intervention, compared with the model group, neuronal morphology and arrangement were restored in the tiapride and CPYJD groups, with the number and staining intensity of cytoplasmic Nissl bodies recovered and the Nissl-positive area significantly increased (*p* < 0.01); the nuclear membrane wrinkling was reduced, the nucleoli became distinct again, nuclear pyknosis or displacement was rarely observed, and the severity of neuronal necrosis and cytoplasmic swelling was alleviated. Compared with the CPYJD-H group, the Nissl-positive areas in the CPYJD-L/M groups and the tiapride group were smaller (*p* < 0.01), indicating that CPYJD exerts a protective effect on neurons in a dose-dependent manner (Figure 2C,D).

### 2.3. CPYJD Ameliorates Disordered Lipid Metabolism in TS Rats

TEM analysis revealed that myelinated nerve fibers in the control group exhibited generally normal ultrastructure, with compact myelin sheaths, parallel lamellar arrangement, no obvious loosened regions, and no apparent axonal atrophy. Compared with the control group, the model group showed severe damage, characterized by shrunken and highly irregular myelin sheaths, demyelination and lamellar splitting, loosely arranged lamellar structures, and disorganized neurofilaments and microtubules that were locally tangled into clusters. Compared with the model group, all treatment groups showed varying degrees of improvement with a dose gradient: the CPYJD-L group still exhibited relatively severe (moderate-to-severe) damage, with thickened and split myelin sheaths, loose and disordered lamellar arrangement, and extensive dissolution and degeneration; the CPYJD-M group showed moderate damage, with focal myelin degeneration, loose and disordered lamellar structures, and blurred structural integrity; the CPYJD-H and tiapride groups showed relatively mild damage, with demyelination largely absent, loosely arranged lamellae and minimal degeneration only in limited areas, and axonal atrophy in some regions (Figure 3A).

Immunohistochemistry (IHC) results demonstrated that compared with the control group, the model group had significantly increased apolipoprotein E (ApoE) expression (*p* < 0.01) and decreased ATP-binding cassette transporter A1 (ABCA1) and ATP-binding cassette subfamily G member 1 (ABCG1) expression (*p* < 0.01). Compared with the model group, the tiapride group showed downregulated ApoE expression (*p* < 0.01) and upregulated ABCA1 and ABCG1 expression (*p* < 0.01). The CPYJD-M and CPYJD-H groups exhibited decreased ApoE expression (*p* < 0.01) and increased ABCA1 and ABCG1 expression (*p* < 0.01). In the CPYJD-L group, ApoE expression was decreased (*p* < 0.05), ABCA1 expression was increased (*p* < 0.01), while no significant difference was detected in ABCG1 expression (*p* > 0.05); compared with the CPYJD-H group, ApoE expression was increased (*p* < 0.05) and ABCA1 and ABCG1 expression was decreased in the CPYJD-L/M and tiapride groups (*p* < 0.01) (Figure 3B,C).

Biochemical analysis of striatal total cholesterol showed that, compared with the control group, the total cholesterol content was significantly increased in the model group (*p* < 0.01). Compared with the model group, the total cholesterol content was decreased in the tiapride and CPYJD-L/M/H groups (*p* < 0.01). Compared with the CPYJD-H group, the total cholesterol content was higher in the CPYJD-L/M groups (*p* < 0.05), while no significant difference was observed in the tiapride group (*p* > 0.05) (Figure 3D).

The mRNA and protein expression of ApoE, ABCA1, and ABCG1 in the striatum was further examined.

Reverse transcription quantitative PCR (RT-qPCR) analysis showed that, compared with the control group, *Apoe* mRNA expression was upregulated (*p* < 0.01), while *Abca1* and *Abcg1* mRNA expression was downregulated in the model group (*p* < 0.01). Compared with the model group, *Apoe* mRNA expression was downregulated (*p* < 0.01) and *Abca1* and *Abcg1* mRNA expression was upregulated in the tiapride group and all CPYJD groups (*p* < 0.01). Compared with the CPYJD-H group, *Apoe* mRNA expression was upregulated (*p* < 0.01) and *Abca1* and *Abcg1* mRNA expression was downregulated in the CPYJD-L group (*p* < 0.01); in the CPYJD-M and tiapride groups, *Abca1* and *Abcg1* mRNA expression was downregulated (*p* < 0.05), while *Apoe* mRNA expression showed no significant difference (*p* > 0.05) (Figure 3E).

Western blot (WB) analysis showed that, compared with the control group, the protein expression of ApoE was upregulated (*p* < 0.01), while that of ABCA1 and ABCG1 was downregulated in the model group (*p* < 0.01). Compared with the model group, ApoE protein expression was downregulated in the tiapride and CPYJD-M/H groups (*p* < 0.01), with no significant difference in the CPYJD-L group (*p* > 0.05); ABCA1 protein expression was upregulated only in the CPYJD-H group (*p* < 0.05), with no significant differences in the CPYJD-L/M or tiapride groups (*p* > 0.05); ABCG1 protein expression was upregulated in the CPYJD-M/H groups (*p* < 0.01), with no significant difference in the CPYJD-L group (*p* > 0.05). Compared with the CPYJD-H group, the CPYJD-L group showed upregulated ApoE protein expression (*p* < 0.01) and downregulated ABCG1 protein expression (*p* < 0.01), with no significant difference in ABCA1 protein expression (*p* > 0.05); no significant differences in the protein expression of ApoE, ABCA1, or ABCG1 were observed in the CPYJD-M or tiapride groups (*p* > 0.05) (Figure 3F,G). These results indicate that CPYJD ameliorates IDPN-induced myelin structural damage and lipid metabolic abnormalities, showing a dose-dependent trend at the mRNA and IHC levels.

### 2.4. CPYJD Regulates Microglial M1-like/M2-like Phenotypic Balance and Alleviates Neuroinflammation in TS Rats

Immunofluorescence (IF) analysis showed that, compared with the control group, ionized calcium-binding adapter molecule 1 (IBA1) expression was increased in the model group (*p* < 0.01). Compared with the model group, IBA1 expression was decreased after intervention in the tiapride group and all CPYJD groups (*p* < 0.01) (Figure 4A,B). Compared with the CPYJD-H group, IBA1 expression was higher in the CPYJD-L/M and tiapride groups (*p* < 0.01). These results indicate that MG were activated after modeling, whereas CPYJD inhibited their excessive activation.

IHC analysis showed that, compared with the control group, the number of inducible nitric oxide synthase (iNOS)-positive cells was increased (*p* < 0.01), while the number of arginase-1 (Arg-1)-positive cells was decreased in the model group (*p* < 0.01). Compared with the model group, the number of iNOS-positive cells was decreased in the tiapride group and all CPYJD groups (*p* < 0.01); the number of Arg-1-positive cells was increased in the tiapride and CPYJD-M/H groups (*p* < 0.01), with no significant difference in the CPYJD-L group (*p* > 0.05). Compared with the CPYJD-H group, the number of iNOS-positive cells was higher in the CPYJD-L/M groups (*p* < 0.01), with no significant difference in the tiapride group (*p* > 0.05); the number of Arg-1-positive cells was lower in the CPYJD-L/M and tiapride groups (*p* < 0.05) (Figure 4C,D).

The mRNA expression of cluster of differentiation 86 (CD86) and cluster of differentiation 206 (CD206) in the striatum was detected by RT-qPCR. The results showed that, compared with the control group, CD86 mRNA expression was upregulated (*p* < 0.01) and CD206 mRNA expression was downregulated (*p* < 0.01) in the model group. Compared with the model group, CD86 mRNA expression was downregulated in the tiapride group and all CPYJD groups (*p* < 0.01); CD206 mRNA expression was upregulated in the tiapride and CPYJD-M/H groups (*p* < 0.01), with no significant difference in the CPYJD-L group (*p* > 0.05). Compared with the CPYJD-H group, CD86 mRNA expression was higher in the CPYJD-L group (*p* < 0.01), with no significant difference in the CPYJD-M or tiapride groups (*p* > 0.05); CD206 mRNA expression was lower in the CPYJD-L/M groups (*p* < 0.05), with no significant difference in the tiapride group (*p* > 0.05) (Figure 4E).

The protein expression of CD86 and CD206 in the striatum was detected by WB. The results showed that, compared with the control group, CD86 protein expression was upregulated (*p* < 0.01) and CD206 protein expression was downregulated (*p* < 0.01) in the model group. Compared with the model group, CD86 protein expression was downregulated (*p* < 0.05) and CD206 protein expression was upregulated (*p* < 0.01) in the tiapride and CPYJD-M/H groups, with no significant differences in CD86 or CD206 protein expression in the CPYJD-L group (*p* > 0.05). Compared with the CPYJD-H group, CD86 protein expression was higher in the CPYJD-L group (*p* < 0.05), with no significant differences in the CPYJD-M or tiapride groups (*p* > 0.05); CD206 protein expression was lower in the CPYJD-L/M groups (*p* < 0.01), with no significant difference in the tiapride group (*p* > 0.05) (Figure 4F,G).

Subsequently, the levels of inflammatory cytokines in the striatum were measured by enzyme-linked immunosorbent assay (ELISA). The results showed that, compared with the control group, the levels of interleukin-1β (IL-1β) and tumor necrosis factor-α (TNF-α) were increased (*p* < 0.01), while those of interleukin-10 (IL-10) and transforming growth factor-β (TGF-β) were decreased in the model group (*p* < 0.01). Compared with the model group, the levels of IL-1β and TNF-α were decreased in the tiapride group and all CPYJD groups (*p* < 0.05); the IL-10 level was increased in the CPYJD-M/H groups (*p* < 0.05), with no significant difference in the CPYJD-L or tiapride groups (*p* > 0.05); the TGF-β level was increased in the tiapride and CPYJD-H groups (*p* < 0.05), with no significant difference in the CPYJD-L/M groups (*p* > 0.05). Compared with the CPYJD-H group, the IL-1β level was higher in the CPYJD-L/M groups (*p* < 0.01), with no significant difference in the tiapride group (*p* > 0.05); the TNF-α level was higher in the CPYJD-L group (*p* < 0.01), with no significant differences in the CPYJD-M or tiapride groups (*p* > 0.05); the levels of IL-10 and TGF-β were lower in the CPYJD-L and tiapride groups (*p* < 0.05), with no significant differences in the CPYJD-M group (*p* > 0.05) (Figure 4H). These results indicate that CPYJD promotes the shift in MG from the M1-like pro-inflammatory phenotype toward the M2-like anti-inflammatory phenotype and attenuates neuroinflammation in TS model rats, with the most pronounced effects observed in the high-dose group.

### 2.5. Effects of CPYJD on the Expression of Factors Related to the TREM2/NF-κB Signaling Axis in TS Rats

To further explore the underlying mechanism, immunofluorescence was performed on the rat striatum. The results showed that, compared with the control group, TREM2 expression was decreased (*p* < 0.01) and transcription factor p65 expression was increased (*p* < 0.01) in the model group. Compared with the model group, TREM2 expression was increased (*p* < 0.05) and p65 expression was decreased (*p* < 0.05) in the tiapride and CPYJD-L/M/H groups. Compared with the CPYJD-H group, TREM2 expression was lower in the CPYJD-L/M and tiapride groups (*p* < 0.05); p65 expression was higher in the CPYJD-L and tiapride groups (*p* < 0.01), with no significant difference in the CPYJD-M group (*p* > 0.05) (Figure 5A,B).

Subsequently, the mRNA expression of *Trem2*, *p65*, and inhibitor of nuclear factor kappa-B alpha (*IκB-α*) was detected by RT-qPCR. The results showed that, compared with the control group, the mRNA expression of *Trem2* and *IκB-α* was downregulated (*p* < 0.01), while that of *p65* was upregulated in the model group (*p* < 0.01). Compared with the model group, *Trem2* mRNA expression was upregulated (*p* < 0.05) and *p65* mRNA expression was downregulated (*p* < 0.01) in the tiapride group and all CPYJD groups; *IκB-α* mRNA expression was upregulated in the CPYJD-M/H and tiapride groups (*p* < 0.05), with no significant difference in the CPYJD-L group (*p* > 0.05). Compared with the CPYJD-H group, *Trem2* mRNA expression was lower in the CPYJD-L and tiapride groups (*p* < 0.01), with no significant difference in the CPYJD-M group (*p* > 0.05); *p65* mRNA expression was higher in the CPYJD-L group (*p* < 0.01), with no significant differences in the CPYJD-M or tiapride groups (*p* > 0.05); *IκB-α* mRNA expression was lower in the CPYJD-L/M and tiapride groups (*p* < 0.01) (Figure 5C).

WB analysis of protein expression showed that, compared with the control group, TREM2 protein expression was downregulated (*p* < 0.01), while p65 protein expression and the p-IκB-α/IκB-α ratio were increased in the model group (*p* < 0.01). Compared with the model group, TREM2 protein expression was upregulated in the tiapride and CPYJD-M/H groups (*p* < 0.01), with no significant difference in the CPYJD-L group (*p* > 0.05); p65 protein expression and the p-IκB-α/IκB-α ratio were decreased in the tiapride group and all CPYJD groups (*p* < 0.01). Compared with the CPYJD-H group, TREM2 protein expression was lower in the CPYJD-L/M and tiapride groups (*p* < 0.01); p65 protein expression was higher in the CPYJD-L/M groups (*p* < 0.01), with no significant difference in the tiapride group (*p* > 0.05); the p-IκB-α/IκB-α ratio was higher in the tiapride group (*p* < 0.01), with no significant difference in the CPYJD-L/M groups (*p* > 0.05) (Figure 5D,E). These results indicate that CPYJD affects the expression of factors related to the TREM2/NF-κB signaling axis in TS rats, and its effects may be associated with the involvement of this signaling axis, with the most pronounced regulatory effects observed in the high-dose group.

### 2.6. Lipidomics Analysis of the Effects of CPYJD on Differentially Expressed Lipids in the Striatum of TS Rats

Given that the CPYJD-H group achieved the optimal therapeutic efficacy in alleviating TS-like symptoms in rats, this dose group was selected for widely targeted lipidomics analysis. Three groups were included in the assay: the control group, model group, and CPYJD-H group, with 5 biological replicates per group. Raw mass spectrometry data were processed using Skyline (version 22.2.0527; MacCoss Lab Software, University of Washington, Seattle, WA, USA) for chromatographic peak extraction and integration, and subsequent data analysis was performed using an in-house R package in R software (version 4.5.1; R Foundation for Statistical Computing, Vienna, Austria). The raw quantitative values were log_2_-transformed, normalized against internal reference markers, and then subjected to median normalization to set the median lipid intensity of each sample to 18. For lipids with missing quantitative values, those missing in more than 30% of the samples were removed, and the remaining missing values were imputed using the k-nearest neighbors (KNN) method. Widely targeted lipidomics analysis identified a total of 1007 lipid species across the three groups. Lipid annotation, functional assignment, and classification were performed using LIPID MAPS (https://www.lipidmaps.org/; accessed 16 July 2025) [29,30,31] (Figure 6A). Principal component analysis (PCA) results showed that PC1 and PC2 explained 22.73% and 16.03% of the total sample variance, respectively. Quality control (QC) samples clustered tightly, indicating reliable data reproducibility. Compared with the control group, the lipid metabolic profile of the model group showed a distinct shift, confirming significant lipid metabolism disorder in model animals. After CPYJD intervention, samples aggregated into an independent cluster with a metabolic profile markedly different from that of the model group, indicating that CPYJD could effectively regulate the disordered lipid metabolism in model rats (Figure 6B).

Differential analysis between groups was performed using Student’s *t*-test to assess the significance of differences, combined with the variable importance in projection (VIP) from the OPLS-DA model for multivariate statistical analysis, and volcano plots were generated. Given that this study is an exploratory lipidomics analysis based on a small sample size with a large number of detected lipids, strict multiple-testing correction would substantially reduce statistical power; therefore, differential lipids were screened using the criteria of raw *p* < 0.05, VIP > 1, and |log_2_FC| > 0, without multiple-testing correction [32,33,34]. The results showed that compared with the control group, 60 lipid species were significantly upregulated and 18 lipid species were significantly downregulated in the model group (Figure 6C). Compared with the model group, 17 lipid species were significantly upregulated and 53 lipid species were significantly downregulated in the CPYJD group (Figure 6D).

Differential lipids were screened using the criteria of *p* < 0.05, VIP > 1, and |log_2_FC| > 0, and heatmaps were generated. The results showed that, compared with the control group, glycerolipids (GL) were the category with the most significant upregulation and the largest number of differentially expressed lipids after modeling, representing the core class driving the lipid profile divergence between the two groups (Figure 7A). Compared with the model group, glycerophospholipids (GP) accounted for the largest proportion of differentially expressed lipids; some abnormally elevated GP species were downregulated, while the expression of some suppressed GP species was restored (Figure 7B).

In-depth analysis of the differentially expressed lipids identified above was performed based on the LIPID MAPS database. Fisher’s exact test was applied to calculate the significance *p*-value to explore whether differentially expressed lipids exhibited a significant enrichment trend in certain functional categories. Functional categories with significant enrichment of differentially expressed lipids were visualized via bubble plots (*p* < 0.05). The results revealed that compared with the control group, the major lipid metabolism abnormalities in the model group were concentrated in the glycerolipid metabolic pathway: triacylglycerols (including ether-modified forms) and monoacylglycerols were massively and abnormally enriched, accompanied by disorders of lysophosphatidylinositol phospholipids (Figure 7C). Compared with the model group, the enrichment level of ether-linked triacylglycerols decreased significantly after CPYJD intervention, indicating that CPYJD could reverse the abnormal accumulation of GL in the model (Figure 7D).

Enrichment analysis of differentially expressed lipids was conducted using LION/web (Lipid Ontology; https://lipidontology.com/; database version: v.2023.04.14; ontology format-version: 1.2; accessed on 16 July 2025) [35,36]. The results showed that, compared with the control group, at the structural level, GL, triacylglycerols [GL0301], and triradylglycerols [GL03] were highly enriched and constituted the core differentially expressed lipids; at the functional level, lipid storage and lipid droplet showed significant enrichment, suggesting substantial triacylglycerol accumulation, dysregulated lipid metabolism, and lipid deposition in the TS model (Figure 7E). CPYJD intervention reversed the lipid storage disorder caused by abnormal triacylglycerol accumulation in the model (Figure 7F).

### 2.7. Screening of the Optimal Intervention Concentration of CPYJD-Medicated Serum

BV2 cells were stimulated with LPS to establish an in vitro inflammatory model, with a control group (normally cultured cells) and groups treated with 5%, 10%, 15%, or 20% (*v*/*v*) CPYJD-medicated serum. Cell viability was measured at 24 and 48 h using the CCK-8 assay. To exclude interference from the serum itself, the cell viability of each medicated serum group was calculated relative to that of the concentration-matched blank rat serum. The results showed that, at both 24 and 48 h, cell viability in the 10% medicated serum group was higher than that in the control and 15% medicated serum groups (*p* < 0.01) (Figure 8A). Meanwhile, nitric oxide (NO) levels were measured using the Griess assay to evaluate the inhibitory effects of different concentrations of medicated serum on LPS-induced inflammatory responses. The results showed that NO levels were significantly increased in the LPS group compared with the control group (*p* < 0.01) and were decreased after CPYJD-medicated serum intervention (*p* < 0.01); no significant difference in NO levels was observed between the 10% medicated serum group and either the control group or the 15% medicated serum group (*p* > 0.05) (Figure 8B). Based on the CCK-8 and Griess assay results, 10% CPYJD-medicated serum was selected for subsequent in vitro experiments.

### 2.8. CPYJD-Medicated Serum Ameliorates Morphological Alterations in BV2 Cells

The morphology of BV2 cells was observed under a light microscope. In the control group, BV2 cells presented round or spindle-shaped somas with an overall resting branched morphology; intercellular boundaries were clear, with no evident somatic swelling or deformation. After LPS intervention, BV2 cells exhibited hypertrophic and rounded somas, with atrophied, shortened, or even absent cellular processes. The cells transformed from a branched phenotype to an amoeboid round shape, which is a typical morphological feature of activated MG, indicating inflammatory activation. CPYJD-medicated serum intervention reversed LPS-induced BV2 cell activation: the degree of somatic hypertrophy was attenuated, branched structures were partially restored, the number of amoeboid cells was significantly reduced, and the proportion of resting branched cells increased. The morphological changes induced by si-TREM2 intervention were consistent with those in the LPS group, and CPYJD-medicated serum also restored the cell morphology following si-TREM2 intervention. These findings suggest that CPYJD-medicated serum inhibited excessive microglial activation (Figure 8C).

### 2.9. Effects of CPYJD-Medicated Serum and TREM2 Knockdown on Lipid Metabolism in LPS-Stimulated BV2 Cells

Lipid droplets in BV2 cells were detected using the BODIPY fluorescent probe. The results showed that, compared with the control group, the mean fluorescence intensity (MFI) of BODIPY was increased in the LPS and si-TREM2 groups (*p* < 0.01); compared with the LPS group, the MFI was decreased in the CPYJD group (*p* < 0.01) and increased in the si-TREM2 group (*p* < 0.01); compared with the si-TREM2 group, the MFI was decreased in the siRNA + CPYJD group (i.e., si-TREM2 + LPS + CPYJD-medicated serum) (*p* < 0.05) (Figure 8D,E).

Biochemical analysis of total cholesterol in BV2 cells showed that, compared with the control group, the total cholesterol content was increased in the LPS and si-TREM2 groups (*p* < 0.01); compared with the LPS group, the total cholesterol content was decreased in the CPYJD group (*p* < 0.05) and increased in the si-TREM2 group (*p* < 0.01); compared with the si-TREM2 group, the total cholesterol content was decreased in the siRNA + CPYJD group (*p* < 0.01) (Figure 8F).

The mRNA expression of *Apoe*, *Abca1*, and *Abcg1* was detected by RT-qPCR. The results showed that, compared with the control group, *Apoe* mRNA expression was upregulated (*p* < 0.01), while *Abca1* and *Abcg1* mRNA expression was downregulated in the LPS and si-TREM2 groups (*p* < 0.01); compared with the LPS group, *Apoe* mRNA expression was downregulated (*p* < 0.01) and *Abca1* and *Abcg1* mRNA expression was upregulated in the CPYJD group (*p* < 0.01); in the si-TREM2 group, *Apoe* mRNA expression was upregulated (*p* < 0.01) and *Abcg1* mRNA expression was downregulated (*p* < 0.05), with no significant difference in *Abca1* mRNA expression (*p* > 0.05); compared with the si-TREM2 group, *Apoe* mRNA expression was downregulated (*p* < 0.01) and *Abca1* and *Abcg1* mRNA expression was upregulated in the siRNA + CPYJD group (*p* < 0.01) (Figure 8G). These results indicate that CPYJD-medicated serum ameliorates lipid metabolism in LPS-stimulated BV2 cells and retains its lipid accumulation-reducing effect even under TREM2 knockdown, suggesting that the regulatory effect of CPYJD on lipid metabolism is associated with TREM2/NF-κB signaling but may not be entirely dependent on TREM2.

### 2.10. Effects of CPYJD-Medicated Serum and TREM2 Knockdown on Microglial Phenotype and Inflammatory Responses in LPS-Stimulated BV2 Cells

Immunofluorescence co-localization analysis showed that, compared with the control group, the MFI of IBA1/iNOS co-localization was increased (*p* < 0.01), while that of IBA1/Arg-1 co-localization was decreased in the LPS and si-TREM2 groups (*p* < 0.01). Compared with the LPS group, the MFI of IBA1/iNOS co-localization was decreased in the CPYJD group (*p* < 0.01) and increased in the si-TREM2 group (*p* < 0.01); the MFI of IBA1/Arg-1 co-localization was increased in the CPYJD group (*p* < 0.01) and decreased in the si-TREM2 group (*p* < 0.05). Compared with the si-TREM2 group, the MFI of IBA1/iNOS co-localization was decreased (*p* < 0.01), while that of IBA1/Arg-1 co-localization was increased in the siRNA + CPYJD group (*p* < 0.01) (Figure 9A,B).

Subsequently, the mRNA expression of cytokines was detected by RT-qPCR. The results showed that, compared with the control group, the mRNA expression of *IL-1β* and *TNF-α* was upregulated (*p* < 0.01), while that of *IL-10* and *TGF-β* was downregulated in the LPS and si-TREM2 groups (*p* < 0.01); compared with the LPS group, the mRNA expression of *IL-1β* and *TNF-α* was downregulated (*p* < 0.01), while that of *IL-10* and *TGF-β* was upregulated in the CPYJD group (*p* < 0.01); in the si-TREM2 group, the mRNA expression of *IL-1β* and *TNF-α* was upregulated (*p* < 0.01), while that of *IL-10* and *TGF-β* was downregulated (*p* < 0.01); compared with the si-TREM2 group, the mRNA expression of *IL-1β* and *TNF-α* was downregulated (*p* < 0.01), while that of *IL-10* and *TGF-β* was upregulated in the siRNA + CPYJD group (*p* < 0.01) (Figure 9C). These results indicate that CPYJD-medicated serum inhibited the LPS-induced M1-like pro-inflammatory phenotype and promoted the shift toward the M2-like anti-inflammatory phenotype in BV2 cells, while downregulating the mRNA expression of pro-inflammatory cytokines and upregulating that of anti-inflammatory cytokines. Under TREM2 knockdown, CPYJD-medicated serum retained the above regulatory effects, suggesting that the effects of CPYJD on microglial phenotype and inflammatory responses are associated with TREM2/NF-κB signaling but may not be entirely dependent on TREM2.

### 2.11. Effects of CPYJD-Medicated Serum on the Expression of Factors Related to the TREM2/NF-κB Signaling Axis in BV2 Cells

To further investigate the underlying mechanism, the mRNA and protein expression of factors related to the signaling axis in BV2 cells was detected by RT-qPCR and WB. Compared with the control group, the mRNA expression of *Trem2* and *IκB-α* was downregulated (*p* < 0.01), while *p65* mRNA expression was upregulated in the LPS and si-TREM2 groups (*p* < 0.01). Compared with the LPS group, the mRNA expression of *Trem2* and *IκB-α* was upregulated (*p* < 0.01), while *p65* mRNA expression was downregulated in the CPYJD group (*p* < 0.01); in the si-TREM2 group, the mRNA expression of *Trem2* and *IκB-α* was downregulated (*p* < 0.01), while *p65* mRNA expression was upregulated (*p* < 0.01). Compared with the si-TREM2 group, the mRNA expression of *Trem2* and *IκB-α* was upregulated (*p* < 0.01), while *p65* mRNA expression was downregulated in the siRNA + CPYJD group (*p* < 0.01) (Figure 10A).

WB analysis showed that, compared with the control group, TREM2 protein expression was downregulated (*p* < 0.01), while p65 protein expression and the p-IκB-α/IκB-α ratio were increased in the LPS and si-TREM2 groups (*p* < 0.05). Compared with the LPS group, TREM2 protein expression was upregulated (*p* < 0.01), while p65 protein expression and the p-IκB-α/IκB-α ratio were decreased in the CPYJD group (*p* < 0.01); in the si-TREM2 group, TREM2 protein expression was downregulated (*p* < 0.01), while p65 protein expression and the p-IκB-α/IκB-α ratio were increased (*p* < 0.05). Compared with the si-TREM2 group, TREM2 protein expression was upregulated (*p* < 0.01), while p65 protein expression and the p-IκB-α/IκB-α ratio were decreased in the siRNA + CPYJD group (*p* < 0.05) (Figure 10B,C). These results indicate that the effects of CPYJD are associated with the TREM2/NF-κB signaling axis.

## 3. Discussion

As a common NDD, TS has attracted widespread attention because of its chronic and recurrent symptoms, which readily impair children’s social functioning. Given the limitations of current behavioral and pharmacological therapies, an increasing number of patients are turning to complementary and alternative medicine (CAM) [37,38]. As an important component of CAM, traditional Chinese medicine (TCM) has gained increasing recognition for its advantages in treating TS. In the present study, we established a TS model by intraperitoneal injection of IDPN. Current rat models of TS mainly focus on striatal disinhibition, which characteristically mirrors the clinical hyperkinesia and abnormal vocalizations, and thus holds broad prospects for future in vivo TS research [39]. The IDPN rat model is a relatively well-established TS model: after successful modeling, rats exhibit a dual behavioral phenotype of increased motor behavior and stereotyped behavior; moreover, it has a short modeling period and low inter-individual variability, and can recapitulate the pathological mechanisms of neurotransmitter abnormalities and neuroinflammation in TS, making it a relatively ideal TS model at present [40,41,42]. Nevertheless, the IDPN-induced TS model has its limitations: as a chemically induced neurotoxic model, it only partially recapitulates the TS phenotype, and vocal tics and premonitory urges are difficult to evaluate in this model; meanwhile, common TS comorbidities, such as attention-deficit hyperactivity disorder and obsessive–compulsive disorder, cannot be reproduced [43,44]. Current pharmacological treatments for TS are mainly divided into three categories: dopamine receptor antagonists, monoamine-depleting agents, and α_2_-adrenergic receptor agonists [45]. Tiapride, a selective striatal D_2_/D_3_ dopamine receptor antagonist, provides marked control of tic symptoms with mild extrapyramidal adverse effects and no significant cardiovascular or systemic metabolic side effects; as a commonly used first-line agent with greater clinical accessibility, it was selected as the positive control drug in this study [7,46].

In the present study, we first identified the major chemical constituents of CPYJD using UPLC-MS/MS. A total of 925 compounds were identified, and the top 20 compounds ranked by peak area were presented, including Parishin E, Orobanone, and Parishin C, which mainly belong to sesquiterpenes and sesquiterpene lactones, phenolic acids and phenolic glycosides, xanthones, triterpenoid sapogenins, nucleosides, oligosaccharides, and amino acid derivatives. Among them, sesquiterpenes, phenolic acids, and phenolic glycosides may constitute the core pharmacodynamic components. It should be noted that this study presents the chemical composition of CPYJD primarily from the perspective of relative content; however, the relative content of a constituent is not equivalent to its contribution to biological activity, and low-abundance constituents may also play critical roles. Moreover, the specific active constituents responsible for TREM2 regulation were not further identified; therefore, the results of this study should be interpreted as the effect of the compound formula as a whole, which should be further verified by formula dissection or constituent-combination experiments in the future.

After modeling, the modeled rats were evaluated according to the criteria for successful model establishment; all modeled rats had motor behavior and stereotyped behavior scores of ≥2 and were eligible for subsequent experiments. The rats were then administered the corresponding drugs for 28 days according to the grouping design. After 28 days of treatment, both tiapride and all CPYJD dose groups effectively reduced the motor behavior and stereotyped behavior scores of TS rats, with the most pronounced improvements observed in the tiapride and CPYJD-H groups. Meanwhile, HE and Nissl staining showed that the CPYJD-M and CPYJD-H groups significantly increased the Nissl-positive area and improved neuronal morphology and attenuated neuronal necrosis and cytoplasmic swelling. The improvement in striatal neuronal morphology is pathophysiologically correlated with the improvement in behavioral scores: clinical studies have confirmed that reduced striatal volume and the selective loss of specific interneuron subpopulations (parvalbumin (PV)-positive and cholinergic interneurons) are associated with tic symptoms in TS patients [47,48]; animal studies have further demonstrated that targeted ablation of dorsal striatal interneurons is sufficient to induce tic-like behaviors, suggesting that impaired structural integrity of striatal neurons may be one of the neural bases underlying tic-like behaviors [49,50]. Therefore, the protective effect of CPYJD on striatal neuronal morphology may serve as the structural basis for the improvement in behavioral scores. However, this study did not perform a correlation analysis between quantitative neuronal morphology and behavioral scores at the individual level, and the quantitative association between the two remains to be verified in future studies. These results indicate that CPYJD effectively ameliorates the symptoms of TS model rats and exerts therapeutic effects. It should be noted that the doses used in this study (19.32, 38.64, and 77.28 g/kg/d) correspond to 1-, 2-, and 4-fold the clinically equivalent dose converted by the body surface area method and are expressed as crude drug equivalents, serving only as the basis for dose conversion and solution preparation; the animals actually received a concentrated aqueous decoction rather than the crude drug itself, and therefore the apparent doses cannot be directly compared with the actual intake of chemical drugs. Accordingly, the therapeutic effects of CPYJD observed in this study are unlikely to be attributable to dose-related nonspecific or toxic effects. First, if CPYJD had exerted nonspecific toxic effects, the expected manifestations would have been deterioration of general condition, aggravated behavioral scores, or elevated inflammation levels; however, the effects observed in this study—including improvement of tic-like behaviors, attenuation of neuroinflammation, and alleviation of striatal pathological damage—were all in the direction consistent with the therapeutic purpose, and the tiapride positive control group showed effects in the same direction. Second, the dose–effect relationship in this study exhibited nonlinear characteristics: some indicators improved with increasing doses, while others reached a plateau after the medium dose, which is inconsistent with the monotonically worsening pattern of nonspecific toxic effects with increasing dose. Nevertheless, the absence of obvious adverse reactions during the 28-day administration period only indicates good short-term tolerability; systematic toxicological evaluation was not performed in this study, and the safety of long-term high-dose CPYJD administration remains to be further verified by objective indicators such as organ coefficients and histopathological toxicity screening.

Myelin, a multilayered membrane structure wrapping neuronal axons, is synthesized by oligodendrocytes in the CNS, and its structural integrity is a prerequisite for normal CNS function [15]. Studies have shown extensive structural abnormalities in the cortico-striato-pallido-thalamic white matter fiber pathways in TS patients [11]. Histidine decarboxylase (*Hdc*) knockout mice exhibit behavioral, brain activity, neurochemical, and neurotransmitter receptor characteristics similar to those of human TS, and diffusion tensor imaging (DTI) has revealed abnormal striatal myelination in these mice [51]. These findings suggest that myelin structural abnormalities may be involved in the pathological process of TS; however, it should be noted that the extent and pathological significance of myelin abnormalities in TS have not been fully established, and the causal relationship between the two remains to be further investigated. In the present study, TEM revealed demyelinating changes in TS model rats, and CPYJD intervention ameliorated myelin damage, suggesting that the protective effect of CPYJD on myelin structure may contribute to its therapeutic effects.

Myelin damage leads to the generation of large amounts of myelin debris. As the resident macrophages of the CNS, MG can clear myelin debris from demyelinating lesions, a process that is a key prerequisite for remyelination [17,52]. However, lipids account for an extremely high proportion (approximately 70%) of myelin content; if the large amounts of lipids produced by microglial degradation of myelin debris cannot be properly metabolized, cholesterol recycling is impaired, ultimately resulting in the accumulation of myelin debris and cholesterol [53]. ApoE, a core transporter of lipids and cholesterol in brain tissue, plays a key regulatory role in lipoprotein metabolism and cholesterol redistribution, with its central function being to mediate lipid efflux and reduce intracellular cholesterol accumulation [54]. The ATP-binding cassette (ABC) transporters ABCA1 and ABCG1 mediate reverse cholesterol transport, promoting intracellular cholesterol efflux and maintaining normal cellular physiological functions [55]. Studies have shown that specific knockout of *Apoe* and the microglial/macrophage lipid efflux genes *Abca1* and *Abcg1* in mice can replicate a pathological model of myelin lipid accumulation [56].

The present study showed that ApoE expression was increased, ABCA1 and ABCG1 expression was decreased, and total cholesterol content was elevated in TS model rats, indicating abnormal lipid transport and lipid accumulation; these alterations were reversed by CPYJD intervention, suggesting that the anti-tic effect of CPYJD is associated with its improvement of lipid transport abnormalities and lipid accumulation. It is worth mentioning that our assessment of cholesterol efflux function was mainly inferred indirectly from the expression changes in ApoE, ABCA1, and ABCG1 together with intracellular total cholesterol content; moreover, BODIPY labels the total pool of neutral lipids, and total cholesterol measurement did not distinguish free cholesterol from cholesteryl esters. Direct functional evidence of cholesterol efflux rates is therefore lacking, which will be further verified in future studies through efflux rate assays and separate quantification of free and esterified cholesterol. Lipidomics results similarly indicated significant lipid metabolism disorders in TS model rats, among which GL constituted the core lipid category disturbed in this model, and GL serves as the major intermediate storage form in myelin lipid recycling. Enrichment analysis further revealed that the differential lipids in model rats were mainly enriched in glycerolipid metabolism pathways (abnormal enrichment of triacylglycerols and monoacylglycerols) and functional terms such as “lipid storage” and “lipid droplet”, suggesting substantial glycerolipid accumulation in the striatum of model rats, which was reversed by CPYJD intervention along with the disordered lipid storage. It should be noted that the purpose of the lipidomics analysis in this study was to explore the metabolic mechanisms underlying the therapeutic effects of CPYJD, rather than to evaluate dose–effect relationships. Based on the previous pharmacodynamic results, the CPYJD-H group (77.28 g/kg/d) showed the most pronounced effects in improving behavioral scores and attenuating neuronal pathological damage; therefore, this dose group was selected for comparison with the model group, in order to capture efficacy-related metabolic changes against the clearest pharmacodynamic background. This strategy of selecting the optimal-effect dose group for omics-based mechanistic exploration is widely adopted in omics studies of TCM compound formulas, and it also effectively controls the cost and sample size of omics detection. However, we acknowledge that including only the high-dose group cannot present the dynamic changes in lipid metabolism with increasing doses, and the metabolic changes observed in this study should be interpreted as the metabolic profile of CPYJD at the optimal-effect dose, rather than complete evidence of dose dependence. Meanwhile, the lipidomics analysis in this study was exploratory and based on a small sample size; differential lipids were screened using the criteria of raw *p* < 0.05, VIP > 1, and |log_2_FC| > 0, which carries a certain risk of false positives, and specific differential lipids could not be directly mapped to the TREM2/NF-κB signaling axis. Future studies will validate the key differential lipids by expanding the sample size, incorporating dose gradients, and performing targeted lipid quantification.

Lipid droplets, organelles that store neutral lipids such as triacylglycerols and cholesteryl esters, are increasingly recognized as hallmark structures of inflammatory responses [55]. Studies have shown that lipid droplet-accumulating microglia exhibit a pro-inflammatory, detrimental activation phenotype [57,58]. RNA sequencing of iMG (induced microglia-like cell) subpopulations revealed that cells with high lipid droplet content exhibited upregulated expression of pro-inflammatory cytokines downstream of NF-κB and downregulated expression of homeostatic microglial signature markers [59]. The NF-κB dimer is composed of p50 and RelA (p65); upon phosphorylation, IκB is degraded, releasing NF-κB, which then translocates from the cytoplasm into the nucleus to activate downstream gene transcription. As a canonical pro-inflammatory signaling pathway, NF-κB regulates the expression of various cytokines and chemokines, including the pro-inflammatory cytokines IL-1β and TNF-α and the anti-inflammatory cytokines IL-10 and TGF-β [60]. The present study showed that microglia in the striatum of TS model rats were in an activated state, with increased IBA1 expression; further investigation of the microglial phenotype revealed increased expression of M1-like pro-inflammatory markers and decreased expression of M2-like anti-inflammatory markers, accompanied by increased pro-inflammatory and decreased anti-inflammatory cytokine levels, suggesting the presence of neuroinflammation associated with the microglial phenotypic shift in the TS model. CPYJD intervention ameliorated excessive microglial activation, promoted the shift from the M1-like pro-inflammatory phenotype toward the M2-like anti-inflammatory phenotype, and suppressed pro-inflammatory cytokine levels while promoting anti-inflammatory cytokine levels, thereby exerting anti-neuroinflammatory effects. However, we acknowledge that this study used iNOS and CD86 as markers of the M1-like phenotype and Arg-1 and CD206 as markers of the M2-like phenotype, and this framework represents a simplification of microglial activation states. Microglial activation exists as a continuous spectrum, with multiple intermediate states and disease-associated subtypes (such as DAM) present in vivo. Moreover, the causal link between lipid accumulation and the microglial phenotypic shift has not been directly demonstrated experimentally. Future studies will employ single-cell RNA sequencing to characterize changes in microglial subpopulations and will functionally validate the direct connection between lipid accumulation and phenotypic shift through exogenous lipid loading, ACAT/SOAT inhibitor intervention, and fluorescence-labeled phagocytosis assays. Further examination of factors related to the TREM2/NF-κB signaling axis revealed that TREM2 expression was downregulated and the NF-κB signaling pathway was activated in TS model rats, whereas CPYJD upregulated TREM2 expression and inhibited the activation of the NF-κB signaling pathway.

Comparative results from the in vivo experiments showed that tiapride, a selective dopamine D_2_/D_3_ receptor antagonist, controls tic symptoms primarily by blocking dopaminergic transmission, with a single target and potentially indirect and limited effects on downstream pathological processes such as lipid metabolism and microglial phenotype. In contrast, CPYJD, as a traditional Chinese medicine formula, acts on pathological processes such as lipid metabolic disturbance and microglial phenotypic shift in a multi-component, multi-target manner; therefore, while achieving behavioral efficacy comparable to that of tiapride, CPYJD exhibited broader regulatory effects on the underlying neuropathological changes. The dose-dependence of CPYJD was observed in behavioral and most molecular indicators, but was not fully consistent across all endpoints. These comparative results suggest that CPYJD possesses relative therapeutic significance by combining symptom control with pathological modulation, providing experimental evidence for its value as a complementary therapeutic strategy for TS.

TREM2, a receptor predominantly expressed on the microglial membrane, is a key molecule regulating the pro-remyelination function of microglia, participating in the regulation of microglial survival, proliferation, and phagocytosis [61]. In *Trem2*-knockdown mice and BV2 cells, microglia fail to upregulate the transcription of genes associated with activation, phagocytosis, and lipid catabolism, accompanied by enhanced NF-κB signaling, NLRP3 inflammasome activation, and downstream inflammatory responses [62]. In the cuprizone-induced chronic demyelination model, *Trem2*-knockout mice exhibit impaired myelin debris clearance, axonal dystrophy, reduced oligodendrocyte numbers, and persistent, unrepaired demyelinating lesions [63]. TREM2 is also a key regulator of cholesterol metabolism, influencing the expression of lipid metabolism-related genes such as *Apoe* and lipoprotein lipase (LPL). Studies have shown that, following 12 weeks of cuprizone-containing diet to establish chronic demyelination, *Trem2*-knockout mice display substantial cholesteryl ester accumulation within microglia, indicating a defective capacity to degrade myelin lipid components and resulting in intracellular lipid overload [64]; after demyelinating injury, abundant myelin debris accumulates within lesions, and defective myelin degradation is observed under TEM [63]. Conversely, activation of TREM2 signaling promotes the translocation of ABCA1 from lysosomes to the plasma membrane in microglia, accelerating cholesterol efflux and reducing intracellular cholesterol and lipid droplet accumulation [65].

In the present study, we knocked down TREM2 in BV2 cells using siRNA to investigate whether the protective effects of CPYJD-medicated serum are associated with TREM2 signaling. Notably, the results of our serum concentration screening showed that cell viability in the 10% CPYJD-medicated serum group was higher than that in the 15% group and the control group, suggesting a possible nonlinear (inverted U-shaped) concentration–effect relationship, a feature consistent with reports in serum pharmacology studies of traditional Chinese medicine compound formulas [66,67]. However, it should be noted that the BV2 immortalized microglial cell line used in our in vitro experiments differs from primary microglia in its biological behavior and lacks the in vivo microenvironment of neuron–glia interactions; therefore, the relevant results should be extrapolated to in vivo settings with caution. The results showed that TREM2-knockdown BV2 cells exhibited more severe lipid transport abnormalities and inflammatory responses, an enhanced shift toward the M1-like pro-inflammatory phenotype, and increased activation of the NF-κB signaling pathway. Intervention with CPYJD-medicated serum attenuated lipid metabolism abnormalities and inflammatory responses in TREM2-knockdown BV2 cells, promoted a shift toward the M2-like anti-inflammatory phenotype, and inhibited the activation of the NF-κB signaling pathway, indicating that the effects of CPYJD-medicated serum on ameliorating lipid metabolism abnormalities and inflammatory responses are associated with the TREM2/NF-κB signaling axis. Although no negative-control siRNA group was included in the siRNA knockdown experiments, which cannot completely exclude non-specific effects of the transfection procedure itself, knockdown efficiency was verified at both mRNA and protein levels, and the phenotypes after knockdown were consistent with the known biological functions of TREM2 and the in vivo results, suggesting that the observed effects mainly arose from specific TREM2 knockdown. In addition, the medicated serum used in the in vitro experiments contains not only CPYJD-derived constituents but also various endogenous serum components; therefore, the observed cellular effects should be interpreted as the overall effect of the medicated serum. Future studies will integrate serum pharmacochemistry, network pharmacology, molecular docking, and single-constituent/constituent-combination validation experiments to systematically elucidate the active constituents of CPYJD responsible for TREM2 regulation and their interaction patterns.

In addition, several limitations of this study should be acknowledged. First, although the TREM2 knockdown experiments provide directional evidence for the relationship among lipid metabolism disorders, microglial phenotypic shift, and TREM2 signaling, siRNA mediated only partial knockdown, and the knockdown experiments were performed solely in vitro, without TREM2 rescue experiments, direct NF-κB intervention, or in vivo pathway validation. Meanwhile, this study adopted a single-endpoint cross-sectional design, which cannot clarify the temporal sequence and causal order between lipid metabolism disorders and TREM2/NF-κB signaling alterations, and the two may also influence each other through a bidirectional regulatory loop. Therefore, the conclusions of this study should be interpreted as an association and partial upstream–downstream relationship between the effects of CPYJD and the TREM2/NF-κB signaling axis; future studies will further elucidate this causal network through TREM2 overexpression/rescue experiments, NF-κB-specific inhibitor intervention, and reverse intervention of lipid metabolism. Second, this study was based on the IDPN-induced TS model, and the observed myelin alterations, lipid metabolism abnormalities, and microglial phenotypic shift can only serve as preliminary evidence at the model level, whose exact significance in human TS remains to be elucidated. To address the limitations of the above model and cellular systems, future studies will validate the key findings using primary microglia, iPSC-derived microglia, and microglia-specific TREM2 knockout animal models. Finally, the assessment of NF-κB pathway activation in this study was mainly based on the p-IκB-α/IκB-α phosphorylation ratio, lacking more direct activation evidence such as p-p65 detection and quantitative analysis of p65 nuclear translocation. In the cellular experiments, ApoE, ABCA1, ABCG1, and inflammatory cytokines were measured only at the mRNA level, and changes at the transcriptional level are not entirely equivalent to changes in protein expression and secretion, although these molecules have been validated at the protein level in animal experiments with consistent trends.

Despite the above limitations, the current findings may provide a basis for future therapeutic research. TREM2 participates in the regulation of microglial metabolism, inflammatory activation, and immune homeostasis, while NF-κB is a core transcriptional regulator of inflammatory responses. The coordinated changes in TREM2 expression, NF-κB activation, lipid metabolism-related indicators, and microglial phenotype observed in this study suggest that restoring microglial lipid metabolism and inflammatory homeostasis may constitute a potential therapeutic strategy for TS. Future studies should clarify whether selective modulation of TREM2 or targeted inhibition of excessive NF-κB activation can reproduce the intervention effects of CPYJD. Moreover, identifying the bioactive constituents of CPYJD that regulate TREM2 and NF-κB signaling may facilitate the development of more targeted interventions for TS.

## 4. Materials and Methods

### 4.1. Animals and Cell Line

Male Sprague-Dawley (SD) rats, aged 3 weeks and weighing 50–70 g, were purchased from Changzhou Cavens Laboratory Animal Co., Ltd. (Changzhou, China) (Certificate No. SCXK (Su) 2021-0013). All rats were housed in the specific pathogen-free (SPF) Experimental Animal Center of Gansu University of Chinese Medicine (Approval No. SYXK (Gan) 2021-0004) under controlled environmental conditions (22–24 °C, 40–50% relative humidity) with a 12 h light/dark cycle. All animals had ad libitum access to standard rodent maintenance diet and ultrapure water throughout the study period. All rats were allowed to acclimate for 7 days before experiments. All experimental protocols were reviewed and approved by the Laboratory Animal Ethics Committee of Gansu University of Chinese Medicine (Approval No. GZRSQ2025-124). This study strictly adhered to all international, national, and institutional guidelines and regulations governing the care and use of laboratory animals. At the end of the experiment, rats were euthanized by cervical dislocation under isoflurane anesthesia (Veterinary Drug Approval No. 020037015; Ringpu (Tianjin) Bio-Pharmacy Co., Ltd., Tianjin, China), and all procedures complied with the euthanasia guidelines of the Laboratory Animal Ethics Committee.

The microglial cell line BV2 was purchased from Wuhan Zishan Biotechnology Co., Ltd. (STCC20009P, Wuhan, China). Cells were cultured in DMEM medium (G4511, Servicebio Technology Co., Ltd., Wuhan, China) supplemented with 10% fetal bovine serum (FBS, G8003, Servicebio Technology Co., Ltd., Wuhan, China) and 1% penicillin-streptomycin solution (G4003, Servicebio Technology Co., Ltd., Wuhan, China). Cells were maintained in a cell culture incubator at 37 °C with 5% CO_2_, with the medium replaced regularly. Subculturing (passaging) and cryopreservation were performed when the cell density reached an appropriate confluence, in order to preserve optimal growth and viability.

### 4.2. Model Establishment

IDPN (W11069, Shanghai Yuanye Bio-Technology Co., Ltd., Shanghai, China) was used in this study, with a density of 1.03 g/mL and a purity of >98%. For preparation of the working solution, 1 mL of the IDPN stock solution was diluted with normal saline to a final volume of approximately 33.65 mL to achieve a final concentration of 30 mg/mL. To establish the TS animal model, IDPN was administered via intraperitoneal (i.p.) injection at a dose of 300 mg/kg per day for 7 consecutive days.

Behavioral assessments were performed on the morning of the day after completion of modeling and on the mornings following treatment days 7, 14, 21, and 28. Each rat was placed in an individual observation cage for a 5 min acclimation period, followed by a 60 min observation period during which scoring was performed every 5 min (12 scores in total). Behavioral scores were recorded independently by two trained observers; for each rat, the mean of the 12 scores was calculated, and the mean values from the two observers were then averaged to obtain the final score for analysis. Testing conditions were kept consistent across all groups to minimize environmental variability. Successful modeling was defined as both motor behavior and stereotyped behavior scores being ≥2. The behavioral scoring criteria are shown in Table 2.

### 4.3. Grouping and Experimental Interventions

After a 1-week acclimation period, SD rats were randomly allocated into six groups (*n* = 12 per group): the control group, model group, tiapride group, CPYJD-L, CPYJD-M, and CPYJD-H groups. With the exception of the control group, all rats underwent TS model induction. After model establishment, the doses were calculated using the adult-to-rat body surface area conversion method. Rats in the tiapride group were administered tiapride (Batch No. DLY24K01, Jiangsu Nhwa Pharmaceutical Co., Ltd., Xuzhou, China) solution at 47.91 mg/kg/d by oral gavage. The clinical daily dose of CPYJD for pediatric patients is 121 g of crude medicinal materials. Based on body surface area conversion, the corresponding adult-equivalent dose is 214.70 g/day. Using an adult-to-rat conversion coefficient of 0.09, the clinical equivalent dose for rats was calculated to be 19.32 g/kg/d. Rats in the low-, medium-, and high-dose CPYJD groups were administered CPYJD at crude drug-equivalent doses of 19.32, 38.64, and 77.28 g/kg/d, respectively, corresponding to 1-, 2-, and 4-fold the clinical equivalent dose. Rats in the control and model groups received ultrapure water at 15 mL/kg by oral gavage. All treatments were administered once daily for 28 consecutive days.

### 4.4. Preparation and Components of CPYJD

CPYJD is composed of Shichangpu (*Acori Tatarinowii Rhizoma*) 10 g (Batch No. 202412006), Yujin (*Curcumae Radix*) 10 g (Batch No. 241101), Yuanzhi (*Polygalae Radix*) 8 g (Batch No. 250316), Chuanniuxi (*Cyathulae Radix*) 10 g (Batch No. 24092901), Tianzhuhuang (*Bambusae Concretio Silicea*) 8 g (Batch No. 251201526), Tianma (*Gastrodiae Rhizoma*) 8 g (Batch No. 20250101), Jiangcan (*Bombyx Batryticatus*) 8 g (Batch No. 250324), Chantui (*Cicadae Periostracum*) 8 g (Batch No. 241101), Quanxie (*Scorpio*) 3 g (Batch No. 24032702), Shijueming (*Haliotidis Concha*) 20 g (Batch No. 230724), Cishi (*Magnetitum*) 20 g (Batch No. 24040101), and Jiaoshanzha (charred *Crataegi Fructus*) 8 g (Batch No. 202501001). All herbal pieces were provided by the TCM Pharmacy of the Affiliated Hospital of Gansu University of Chinese Medicine (Lanzhou, China) and complied with the standards of the 2025 edition of the Pharmacopoeia of the People’s Republic of China. One dose of the CPYJD prescription, totaling 121 g of crude drugs, was decocted three times with 10-, 8-, and 6-fold volumes of water for 90, 60, and 30 min, respectively. The three decoctions were combined and concentrated under reduced pressure using a rotary evaporator to 23.486 mL. The resulting concentrate had a crude drug equivalent concentration of approximately 5.152 g/mL, which was essentially consistent with the concentration calculated for the high-dose group based on the administration dose and gavage volume. The solutions for the low- and medium-dose groups were prepared by diluting the high-dose solution with ultrapure water to crude drug equivalent concentrations of 1.288 and 2.576 g/mL, respectively.

The chemical profile of CPYJD was characterized by UPLC-MS/MS. Briefly, 1 mL of CPYJD sample was transferred to a centrifuge tube, followed by the addition of 2 volumes of extraction solvent (methanol:acetonitrile = 1:1, *v*/*v*). After vortexing for 60 s, the mixture underwent low-temperature ultrasonic extraction for 30 min, and was then centrifuged at 12,000 rpm and 4 °C for 10 min. The supernatant was collected and incubated at −20 °C for 1 h to precipitate proteins. After a second centrifugation at 12,000 rpm and 4 °C for 10 min, the supernatant was vacuum-dried. The dried residue was reconstituted with 100 μL of 50% acetonitrile, fully vortexed, and centrifuged again at 12,000 rpm and 4 °C for 10 min. The final supernatant was collected for instrumental analysis. Chromatographic separation and mass spectrometric detection were performed on a Vanquish ultra-high-performance liquid chromatography system (Thermo Fisher Scientific, Waltham, MA, USA) coupled with a Q Exactive HFX high-resolution mass spectrometer (Thermo Fisher Scientific, Waltham, MA, USA). Compounds were identified by combining MS1 and MS2 information: candidate compounds were first screened with a precursor mass error tolerance of ±5 ppm; MS/MS fragmentation spectra were then matched against an in-house traditional Chinese medicine MS/MS spectral database combined with known fragmentation patterns, and identifications with a match score > 0.5 (out of 1.0) were considered reliable. According to the confidence levels defined by the Metabolomics Standards Initiative (MSI), the identifications in this study correspond to putative annotations based on spectral library matching (Level 2/3) and were not individually confirmed by authentic reference standards.

### 4.5. Broad-Targeted Lipidomics Analysis

Striatal tissues were collected from 5 rats in each of the control group, model group, and CPYJD-H group for broad-targeted lipidomics analysis. Briefly, 5 mg of each striatal sample was pulverized with a mortar and transferred to a 2 mL Eppendorf (EP) tube. A total of 200 μL of extraction solution (prepared with 80% methanol) and 2 μL of mixed internal standard solution were added, followed by vortexing for 1 min. Then 960 μL of methyl tert-butyl ether (MTBE) was added and vortexed for 1 min, after which 240 μL of deionized water was added and vortexed for an additional 2 min. The mixture was centrifuged at 10,000 × g for 10 min until clear phase separation was observed. A 600 μL aliquot of the upper organic phase was transferred to a new 2 mL EP tube, dried under a nitrogen stream or via vacuum concentration, and stored at −80 °C. Chromatographic separation of target compounds was performed on a Vanquish Flex UHPLC system (Thermo Fisher Scientific, Waltham, MA, USA) using a Thermo Accucore™ C18 HPLC column (2.1 mm × 100 mm, 2.6 μm particle size). Mobile phase A consisted of 50% water and 50% acetonitrile supplemented with 10 mmol/L ammonium formate; mobile phase B was composed of 10% acetonitrile, 88% isopropanol and 2% water, also containing 10 mmol/L ammonium formate. Chromatographic peak extraction and integration were performed using Skyline software (version 22.2.0527; MacCoss Lab Software, University of Washington, Seattle, WA, USA), and subsequent data analysis was completed with a self-developed R package in R software (version 4.5.1; R Foundation for Statistical Computing, Vienna, Austria). All detected lipids were annotated, functionally defined and classified based on LIPID MAPS (https://www.lipidmaps.org/; accessed on 16 July 2025) and LION/web (Lipid Ontology; http://lipidontology.com/; accessed on 16 July 2025). Visualization analysis was conducted using the ggplot2 package (version 3.4.4).

### 4.6. Histopathology

At 24 h after the last administration, rats in each group were anesthetized with isoflurane and transcardially perfused with 4% paraformaldehyde. Brain tissues were collected and fixed in the same fixative for 24 h. The tissues were then washed, dehydrated, embedded in paraffin, sectioned (4 μm), deparaffinized, and rehydrated, followed by HE staining and Nissl staining, respectively. The sections were mounted and observed under an upright light microscope (NIKON ECLIPSE E100, Nikon, Tokyo, Japan), and images were acquired using a digital slide scanner (LG-FS80, Servicebio, Wuhan, China). Image analysis was performed using CaseViewer 2.4 software (3DHISTECH, Budapest, Hungary). The Nissl-positive area was quantified using Fiji (ImageJ) software (version 1.54p, National Institutes of Health, Bethesda, MD, USA).

### 4.7. Transmission Electron Microscopy (TEM)

Striatal tissue blocks of approximately 1 mm^3^ were placed in EP tubes containing electron microscopy fixative and stored at 4 °C. After rinsing three times with 0.1 M phosphate buffer (PB), the samples were post-fixed with 1% osmium tetroxide and dehydrated through a graded series of ethanol and acetone. The tissues were then embedded in epoxy resin, and ultrathin sections of approximately 70 nm were prepared. After double staining with 2% uranyl acetate and 2.6% lead citrate solutions, the ultrastructural changes were observed under a transmission electron microscope (HT7800, Hitachi, Tokyo, Japan).

### 4.8. Immunofluorescence (IF) and BODIPY Staining

Paraffin sections of rat brain tissue were deparaffinized and rehydrated. Antigen retrieval was performed using citrate antigen retrieval solution (20×, pH 6.0) diluted to 1×, followed by blocking of endogenous peroxidase activity with 3% hydrogen peroxide and blocking of non-specific binding sites with 10% BSA. The sections were incubated overnight at 4 °C with rabbit primary antibodies against IBA1 (GB153502, Servicebio, 1:500), TREM2 (83438-6-RR, Proteintech, Wuhan, China, 1:2000), and p65 (GB11997, Servicebio, 1:1000), followed by HRP-conjugated goat anti-rabbit secondary antibody (GB23303, Servicebio, 1:1000) for 50 min at room temperature. Signal amplification was performed using TSA reagent, and nuclei were counterstained with DAPI. For cell climbing slides, the cells were permeabilized with 0.3% permeabilization solution or left unpermeabilized depending on whether the target protein was a membrane protein, blocked with 10% BSA, and incubated with rabbit primary antibodies against iNOS (GB11119, Servicebio, 1:1000) and Arg-1 (GB15285, Servicebio, 1:1000), with subsequent steps as described above. Intracellular neutral lipids were stained with BODIPY 493/503 fluorescent probe (G1905, Servicebio): the lipid droplet detection probe and detection buffer were thoroughly mixed at a ratio of 1:200 to prepare the BODIPY working solution, which was added to the histochemistry circle and incubated for 30 min at room temperature, and nuclei were counterstained with DAPI. After mounting with anti-fade mounting medium, images were acquired under an upright fluorescence microscope (Eclipse C1, Nikon). The MFI was measured using Fiji (ImageJ) software.

### 4.9. Immunohistochemistry (IHC)

Paraffin sections of rat brain tissue were deparaffinized and rehydrated. Antigen retrieval was performed using citrate antigen retrieval solution (20×, pH 6.0) diluted to 1×, followed by blocking of endogenous peroxidase activity with 3% hydrogen peroxide and blocking of non-specific binding sites with 10% BSA. The sections were incubated overnight at 4 °C with rabbit primary antibodies against ApoE (GB115743, Servicebio, 1:1000), ABCA1 (26564-1-AP, Proteintech, 1:1000), ABCG1 (13578-1-AP, Proteintech, 1:1000), iNOS (GB11119, Servicebio, 1:1000), and Arg-1 (GB15285, Servicebio, 1:1000), followed by HRP-conjugated goat anti-rabbit secondary antibody (GB23303, Servicebio, 1:1000) for 50 min at room temperature. Color development was performed using DAB chromogen, with positive signals appearing brownish-yellow. Nuclei were counterstained with hematoxylin, and the sections were dehydrated through a graded series, mounted, and observed under an upright light microscope (E100, Nikon). The positive area was quantified using Fiji (ImageJ) software.

### 4.10. Automated Biochemical Assay

Rat striatal tissues and BV2 cells were collected, and a chloroform–methanol mixture (2:1, *v*/*v*) was added at the appropriate ratio, followed by mechanical homogenization in an ice bath. After centrifugation, the supernatant was collected. Total cholesterol content was measured using an automated biochemical analyzer (Chemray800, Rayto, Shenzhen, China) according to the instructions of the total cholesterol assay kit (S03042, Rayto).

### 4.11. Cytokine Detection by Enzyme-Linked Immunosorbent Assay (ELISA)

After anesthesia, blood was collected from the abdominal aorta of rats, and serum was separated by centrifugation at 3000 rpm for 10 min. According to the manufacturers’ instructions, commercial ELISA kits were used to measure the levels of IL-1β (GER0002, Servicebio), TNF-α (GZER0004, Servicebio), IL-10 (GER0003, Servicebio), and TGF-β (GER0051, Servicebio) in serum, and data were acquired using a multi-mode microplate reader (SpectraMax i3x, Molecular Devices, San Jose, CA, USA).

### 4.12. RT-qPCR Detection

A total of 20 mg of rat striatal tissue and BV2 cells were collected, and total RNA was extracted according to the instructions of the total RNA extraction kit (19221ES60, Yeasen Biotechnology, Shanghai, China). Briefly, 1 mL of RNA extraction reagent was added, and tissues were ground or cells were disrupted by pipetting, followed by centrifugation to precipitate RNA. RNA concentration was then measured, and RNA was reverse-transcribed into cDNA according to the instructions of the reverse transcription kit (11141ES60, Yeasen). Using GAPDH as the internal reference, amplification was performed with SYBR Green Master Mix (11184ES03, Yeasen) on an RT-qPCR instrument (LightCycler 96, Roche, Basel, Switzerland), and the relative mRNA expression levels were calculated using the 2^−ΔΔCt^ method. The primer sequences are shown in Table 3.

### 4.13. Western Blot

Striatal tissues were placed in grinding tubes with grinding beads, and RIPA lysis buffer (G2002, Servicebio) was added at a ratio of 1:10; the effective lytic components of the buffer were 1% Triton X-100, 1% sodium deoxycholate, and 0.1% SDS. Cells were collected into centrifuge tubes after trypsin digestion, centrifuged at 1000 rpm for 5 min, and the cell pellets were collected and lysed with an appropriate volume of lysis buffer. After grinding, lysis, and centrifugation, the supernatant was collected, and protein concentration was determined using a BCA protein assay kit (G2026, Servicebio), followed by denaturation at 100 °C for 10 min. Subsequently, 10% SDS-PAGE gels were prepared, and all lanes were loaded with 5 μL of sample and electrophoresed at a constant voltage of 150 V until the bromophenol blue migrated to approximately 1 cm from the bottom of the gel. Proteins were then transferred to PVDF membranes at a constant current of 350 mA for 30 min. After blocking with protein-free rapid blocking buffer (G2052, Servicebio) for 30 min at room temperature, the membranes were incubated overnight at 4 °C with the following primary antibodies: ApoE (GB115743, Servicebio, 1:1000), CD86 (GB155630, Servicebio, 1:1000), CD206 (GB153497, Servicebio, 1:1000), p65 (GB11997, Servicebio, 1:1000), β-actin (GB15003, Servicebio, 1:5000), ABCA1 (26564-1-AP, Proteintech, 1:1000), ABCG1 (13578-1-AP, Proteintech, 1:1000), p-IκB-α (82349-1-RR, Proteintech, 1:2000), IκB-α (10268-1-AP, Proteintech, 1:1000), and TREM2 (83438-6-RR, Proteintech, 1:2000). After washing with TBST, HRP-conjugated goat anti-rabbit secondary antibody (GB23303, Servicebio, 1:3000) was applied for 30 min at room temperature. Protein bands were visualized using enhanced ECL chemiluminescence reagent (G2020, Servicebio) and captured with a chemiluminescence imaging system (GelView 6000 Plus, iBIOGENE, Beijing, China). Band gray values were analyzed using Fiji (ImageJ) software.

### 4.14. Preparation of CPYJD-Medicated Serum

Thirty SD rats were randomly divided into a blank serum group and a CPYJD-medicated serum group (*n* = 15 per group). The CPYJD-medicated serum group was intragastrically administered CPYJD at a dose of 77.28 g/kg/d (solution concentration: 5.152 g/mL), while the blank serum group received an equal volume of ultrapure water. Administration was performed twice daily (morning and evening, 12 h apart) for 7 consecutive days. One hour after the final administration, rats were anesthetized by isoflurane inhalation, and blood was collected from the abdominal aorta, followed by centrifugation at 3000 rpm for 10 min. The supernatant was collected, incubated in a 56 °C water bath for 30 min to inactivate complement, sterilized by filtration through a 0.22 μm membrane, aliquoted into 2 mL EP tubes, and stored at −80 °C until use.

### 4.15. Cell Model Establishment and TREM2 Knockdown

The cell experiments were divided into five groups: the control group (normally cultured cells), the LPS group (LPS stimulation), the CPYJD group (LPS + 10% CPYJD-medicated serum), the si-TREM2 group (si-TREM2 transfection + LPS stimulation), and the siRNA + CPYJD group (si-TREM2 transfection + LPS stimulation + 10% CPYJD-medicated serum). All siRNA mentioned herein refers to TREM2-targeting siRNA (si-TREM2). Except for the control group, all groups were treated with 1 μg/mL LPS (L8880, Solarbio Science & Technology Co., Ltd., Beijing, China) for 12 h to establish the BV2 cell polarization model.

si-TREM2 was designed and synthesized by GenePharma Co., Ltd. (Shanghai, China). Briefly, 17 μL of buffer was placed in a sterile RNase-free EP tube, followed by the addition of 30 pmol siRNA with gentle mixing by pipetting. Then, 3 μL of siRNA-mate plus transfection reagent was added and mixed thoroughly before being applied to the cells for culture.

### 4.16. CCK-8 Assay for Cell Viability

BV2 cells were seeded into 96-well plates at 5 × 10^3^ cells per well. After cell adherence, CPYJD-medicated serum at volume fractions of 5%, 10%, 15%, and 20% was added, and the cells were treated for 24 h or 48 h. For each medicated serum group, blank serum at the same final volume fraction was set as a reference to exclude the influence of the serum matrix itself on cell viability. After the intervention, 10 μL of CCK-8 solution (CA1210, Solarbio) was added to each well, followed by incubation at 37 °C for 1 h. The absorbance at 450 nm was then measured using a microplate reader. Cell viability (%) = (OD of the medicated serum group − OD of the blank well)/(OD of the blank serum group at the same concentration − OD of the blank well) × 100%, which was used to evaluate cell viability and screen for the optimal intervention concentration and duration.

### 4.17. Griess Assay for Nitric Oxide (NO) Determination

BV2 cells were seeded into 6-well plates at 2.5 × 10^4^ cells per well. After 24 h, the cells were pre-treated with CPYJD-medicated serum at volume fractions of 5%, 10%, 15%, and 20% for 2 h, followed by the addition of 1 μg/mL LPS for 12 h to establish the model. The cell culture supernatants were collected into 5 mL centrifuge tubes and centrifuged at 1000 rpm for 5 min, and the supernatants were transferred to EP tubes. NO levels were measured using an NO assay kit (S0020S, Beyotime, Shanghai, China). Briefly, 50 μL of sample, Griess Reagent I, and Griess Reagent II were sequentially added to a 96-well plate, and the absorbance was measured at 540 nm. The NO concentration in the samples was calculated according to the standard curve.

### 4.18. Statistical Analysis

In the histological and molecular biological analyses of animal experiments, *n* represents the number of biologically independent animals per group; in cell experiments, *n* represents the number of independent experimental replicates. Data were analyzed using SPSS 26.0 statistical software (IBM Corp., Armonk, NY, USA). Measurement data conforming to a normal or approximately normal distribution are expressed as the mean ± standard deviation ($\bar{x}$ ± s). Intergroup comparisons were performed using one-way analysis of variance (one-way ANOVA), followed by the LSD test for pairwise comparisons when variances were homogeneous, or Dunnett’s T3 test when variances were heterogeneous. A *p* < 0.05 was considered statistically significant. For lipidomics, differential lipids were screened using the criteria of VIP > 1 from the OPLS-DA model, a raw *p* < 0.05, and |log_2_FC| > 0, without multiple testing correction. Graphs were generated using GraphPad Prism 10.0 software (GraphPad Software Inc., San Diego, CA, USA).

## 5. Conclusions

In summary, through in vivo and in vitro experiments combined with lipidomics analysis, this study suggests that lipid metabolic disturbance, together with the phenotypic shift in MG and increased inflammatory cytokine levels, may be involved in the pathological process of TS, in which the TREM2/NF-κB signaling axis may play an important role. In TS model rats, CPYJD alleviated pathological injury of striatal neurons, improved lipid metabolic abnormalities and lipid accumulation in MG, promoted a shift in MG from the M1-like pro-inflammatory phenotype toward the M2-like anti-inflammatory phenotype, and attenuated neuroinflammation; these effects may be associated with the involvement of the TREM2/NF-κB signaling axis (Figure 11). In LPS-stimulated BV2 cells, CPYJD-medicated serum similarly improved lipid metabolism, regulated the microglial phenotype, and suppressed inflammatory responses. TREM2 knockdown aggravated the aforementioned pathological changes, and the effects of CPYJD were partially attenuated but retained after TREM2 knockdown, suggesting that its action is partially dependent on TREM2. It should be noted that the current evidence is limited to animal models and the BV2 cell line, and the proposed mechanism remains an inference based on correlative evidence, which warrants further verification. This study enriches our understanding of the pathological mechanisms underlying TS and provides an experimental basis for future research on CPYJD; its translational value remains to be confirmed in primary MG, additional animal models, and clinical studies.

## Figures and Tables

**Figure 1:**
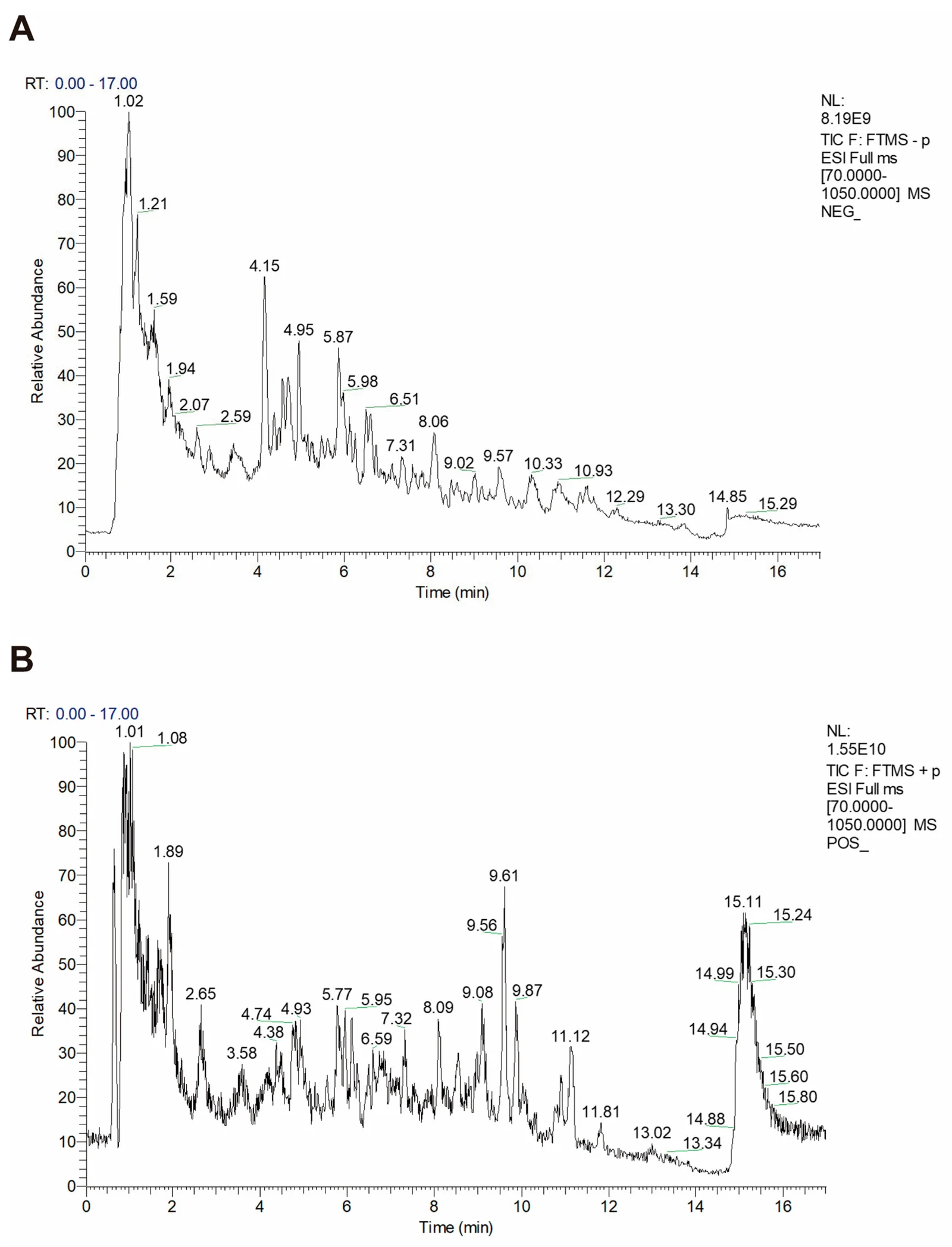
Total ion chromatograms (TICs) of CPYJD acquired by UPLC-MS/MS. (**A**) TIC profile acquired in positive-ion mode; (**B**) TIC profile acquired in negative-ion mode. Chromatographic separation was performed on a Waters HSS T3 column (100 mm × 2.1 mm, 1.8 μm particle size). The mobile phase consisted of ultrapure water containing 0.1% formic acid (phase A) and acetonitrile containing 0.1% formic acid (phase B), delivered at a flow rate of 0.3 mL/min. The column temperature was maintained at 40 °C, and the injection volume was 2 μL. Key mass spectrometry parameters were set as follows: spray voltage of +3000 V/−2800 V, ion transfer tube temperature of 320 °C, and full MS/data-dependent tandem MS acquisition (Full MS-ddMS^2^) performed in both positive and negative ionization modes.

**Figure 2:**
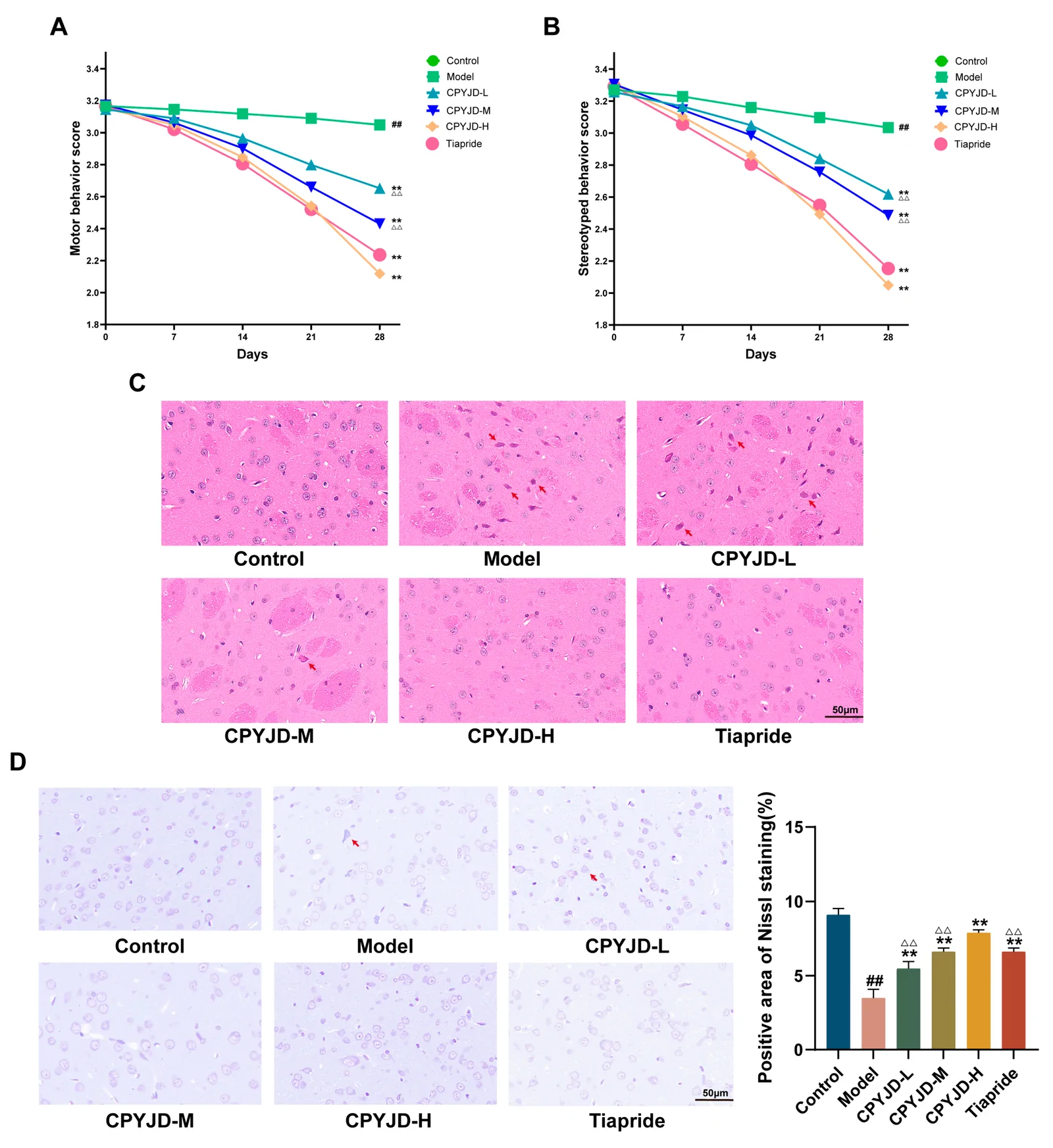
CPYJD improves behavioral scores and neuronal pathological manifestations in TS rats. (**A**) Motor behavior score (*n* = 12 rats per group; Dunnett’s T3 post-hoc test). (**B**) Stereotyped behavior score (*n* = 12 rats per group; Dunnett’s T3 post-hoc test). (**C**) HE staining of the striatum (*n* = 3 rats per group). (**D**) Nissl staining and quantification of the Nissl-positive area in the striatum (*n* = 3 rats per group; LSD post-hoc test). Magnification × 200. Data are expressed as mean ± SD. # *p* < 0.05, ## *p* < 0.01 vs. control group; * *p* < 0.05, ** *p* < 0.01 vs. model group; △ *p* < 0.05, △△ *p* < 0.01 vs. CPYJD-H group.

**Figure 3:**
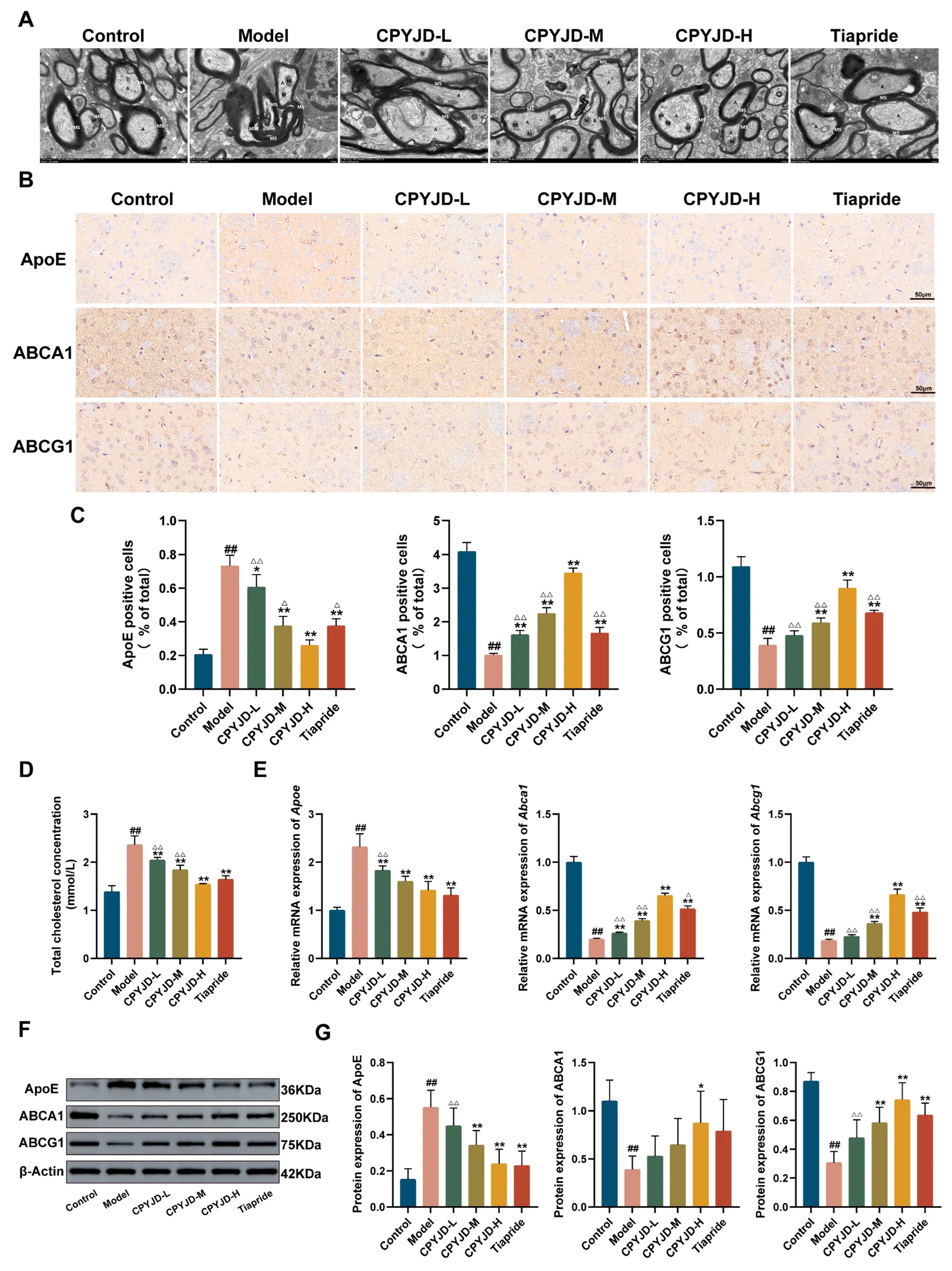
CPYJD ameliorates disordered lipid metabolism in TS rats. (**A**) TEM of myelin alterations in the striatum (*n* = 3 rats per group, magnification × 5000, scale bar = 2 μm); MS denotes myelin sheath, A denotes axon, and M denotes mitochondrion. (**B**) IHC detection of ApoE, ABCA1, and ABCG1 expression in the striatum (*n* = 3 rats per group, magnification × 200). (**C**) Quantification of ApoE-, ABCA1-, and ABCG1-positive cells (*n* = 3 rats per group; LSD post-hoc test). (**D**) Total cholesterol concentration in the striatum (*n* = 3 rats per group; LSD post-hoc test). (**E**) Relative mRNA expression of *Apoe*, *Abca1*, and *Abcg1* in the striatum (*n* = 3 rats per group; LSD, Dunnett’s T3, and LSD post-hoc tests, respectively). (**F**) Representative Western blot bands of ApoE, ABCA1, and ABCG1 (*n* = 3 rats per group). (**G**) Quantification of protein expression of ApoE, ABCA1, and ABCG1 in the striatum (*n* = 3 rats per group; LSD post-hoc test). Data are expressed as mean ± SD. # *p* < 0.05, ## *p* < 0.01 vs. control group; * *p* < 0.05, ** *p* < 0.01 vs. model group; △ *p* < 0.05, △△ *p* < 0.01 vs. CPYJD-H group.

**Figure 4:**
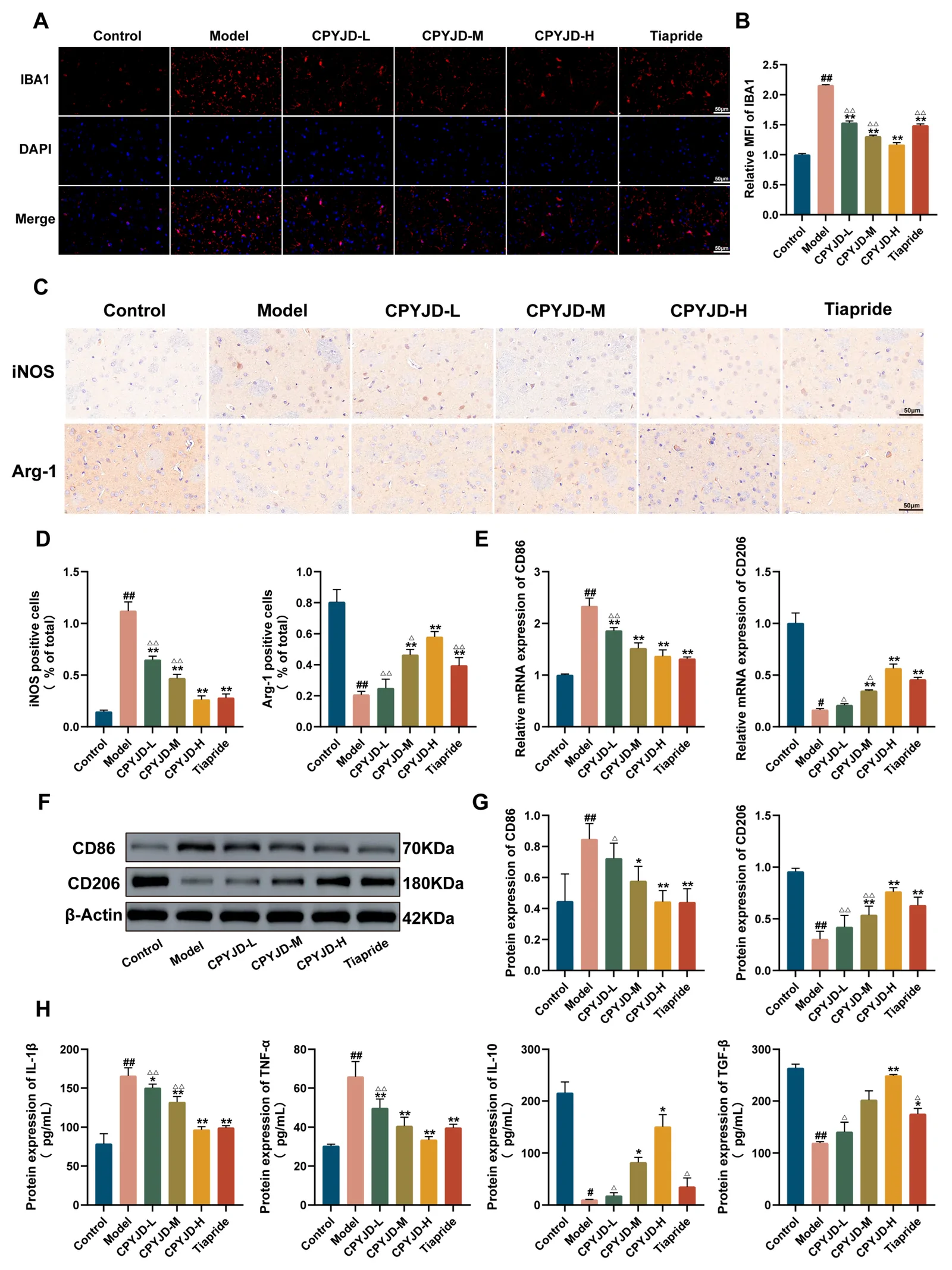
CPYJD regulates microglial M1-like/M2-like phenotypic balance and alleviates neuroin flammation in TS rats. (**A**) Immunofluorescence detection of IBA1 expression in the striatum (*n* = 3 rats per group). (**B**) Quantification of the mean fluorescence intensity (MFI) of IBA1 in the striatum (*n* = 3 rats per group; LSD post-hoc test). (**C**) IHC detection of iNOS and Arg-1 expression in the striatum (*n* = 3 rats per group). (**D**) Quantification of iNOS- and Arg-1-positive cells (*n* = 3 rats per group; LSD post-hoc test). (**E**) Relative mRNA expression of CD86 and CD206 in the striatum (*n* = 3 rats per group; LSD and Dunnett’s T3 post-hoc tests, respectively). (**F**) Representative Western blot bands of CD86 and CD206 (*n* = 3 rats per group). (**G**) Quantification of protein expression of CD86 and CD206 in the striatum (*n* = 3 rats per group; LSD post-hoc test). (**H**) Levels of IL-1β, TNF-α, IL-10, and TGF-β in the striatum measured by ELISA (*n* = 3 rats per group; LSD, LSD, Dunnett’s T3, and Dunnett’s T3 post-hoc tests, respectively). Magnification × 200. Data are expressed as mean ± SD. # *p* < 0.05, ## *p* < 0.01 vs. control group; * *p* < 0.05, ** *p* < 0.01 vs. model group; △ *p* < 0.05, △△ *p* < 0.01 vs. CPYJD-H group.

**Figure 5:**
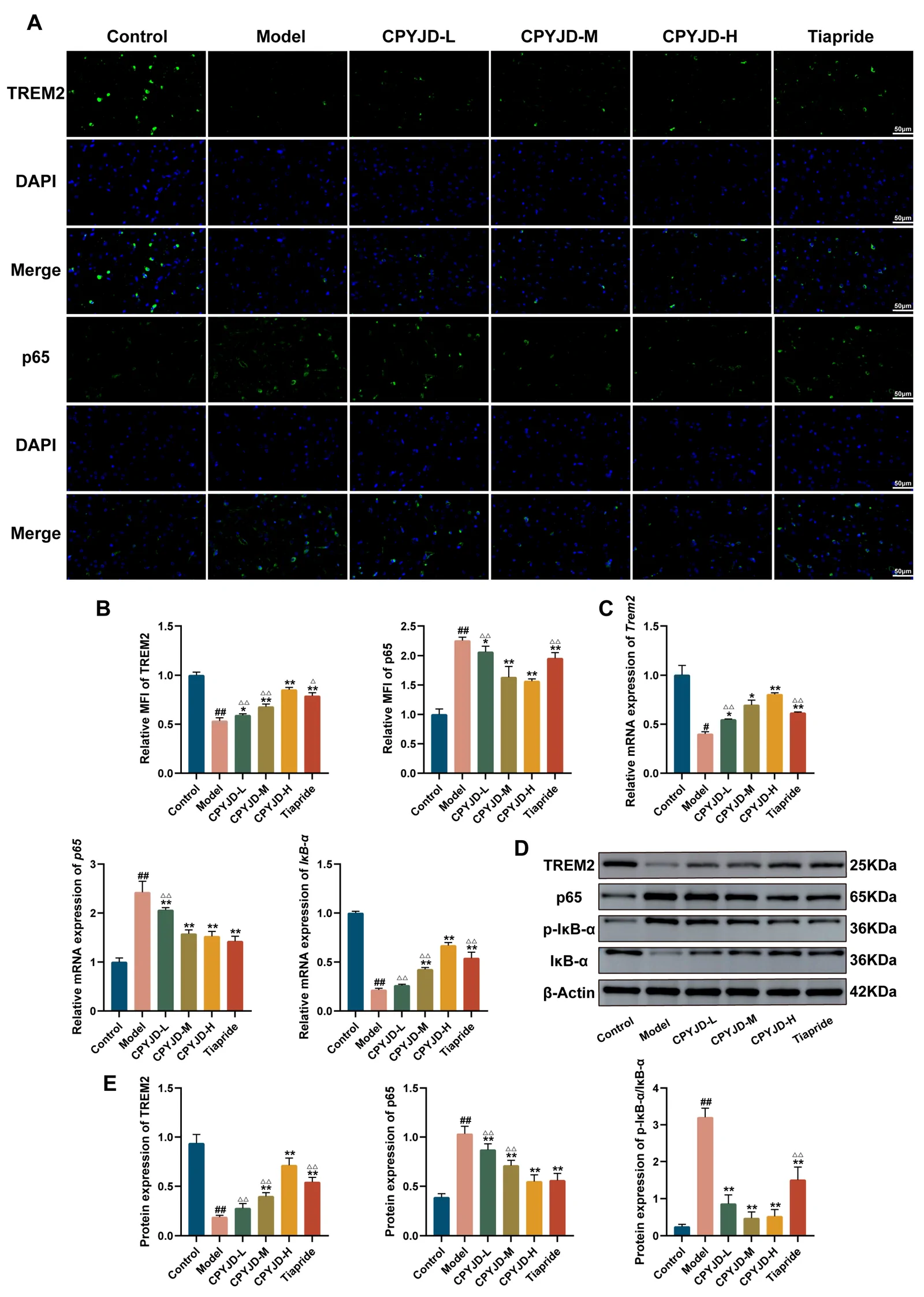
Effects of CPYJD on the expression of factors related to the TREM2/NF-κB signaling axis in TS rats. (**A**) Immunofluorescence detection of TREM2 and p65 expression in the striatum (*n* = 3 rats per group). (**B**) Quantification of the MFI of TREM2 and p65 in the striatum (*n* = 3 rats per group; LSD post-hoc test). (**C**) Relative mRNA expression of *Trem2*, *p65*, and *IκB-α* in the striatum (*n* = 3 rats per group; Dunnett’s T3, LSD, and LSD post-hoc tests, respectively). (**D**) Representative Western blot bands of TREM2, p65, IκB-α, and p-IκB-α (*n* = 3 rats per group). (**E**) Quantification of protein expression of TREM2 and p65, and the p-IκB-α/IκB-α ratio in the striatum (*n* = 3 rats per group; LSD post-hoc test). Magnification × 200. Data are expressed as mean ± SD. # *p* < 0.05, ## *p* < 0.01 vs. control group; * *p* < 0.05, ** *p* < 0.01 vs. model group; △ *p* < 0.05, △△ *p* < 0.01 vs. CPYJD-H group.

**Figure 6:**
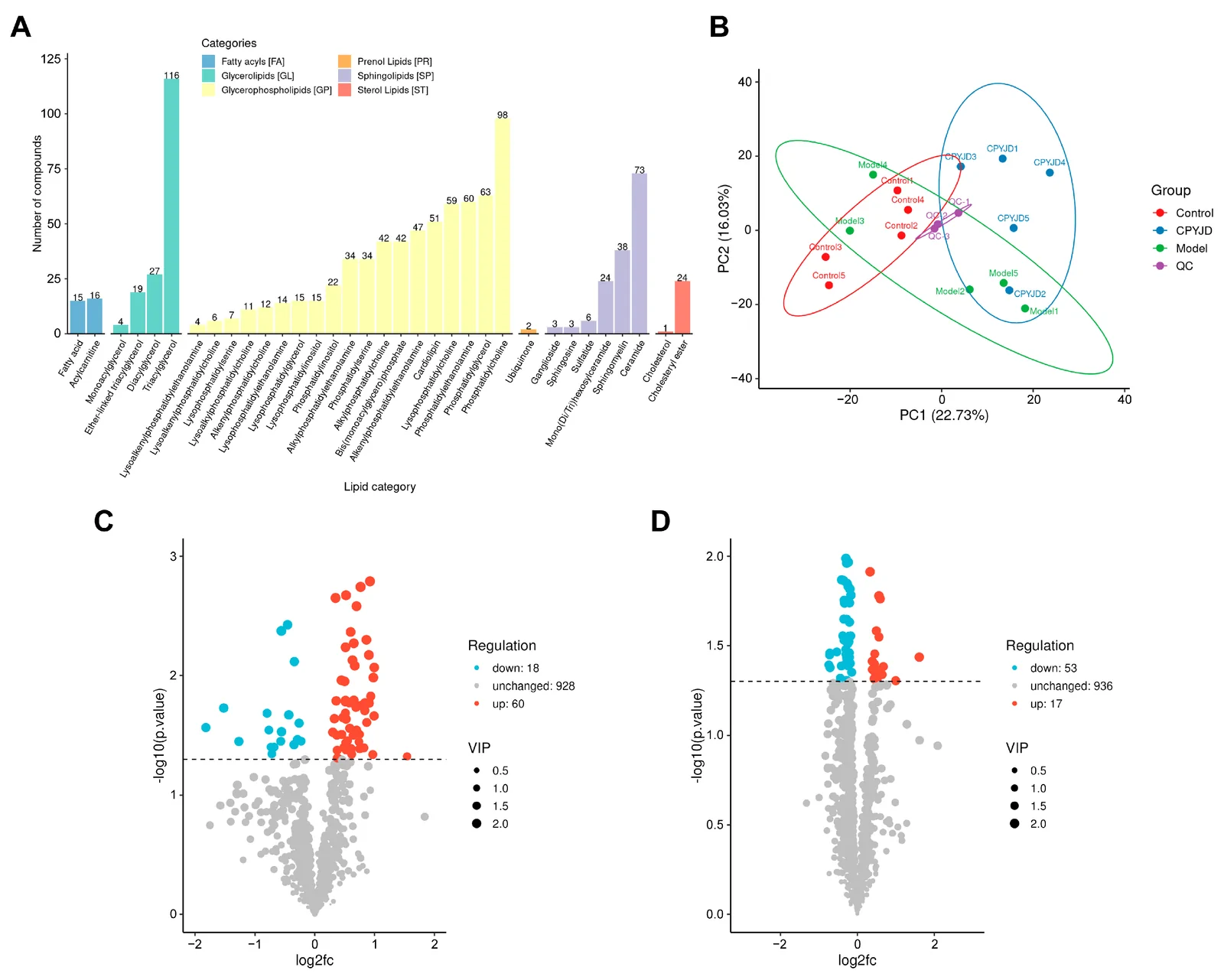
Lipidomics analysis of the effects of CPYJD on differentially expressed lipids in the striatum of TS rats. (**A**) LIPID MAPS database was applied to define the biological functions of identified lipids (*n* = 5 rats per group). (**B**) Principal component analysis (PCA) results based on lipid quantification of all samples (*n* = 5 rats per group). (**C**) Volcano plot for analysis of differentially expressed lipids between control and model groups (*n* = 5 rats per group). (**D**) Volcano plot for analysis of differentially expressed lipids between model and CPYJD groups (*n* = 5 rats per group).

**Figure 7:**
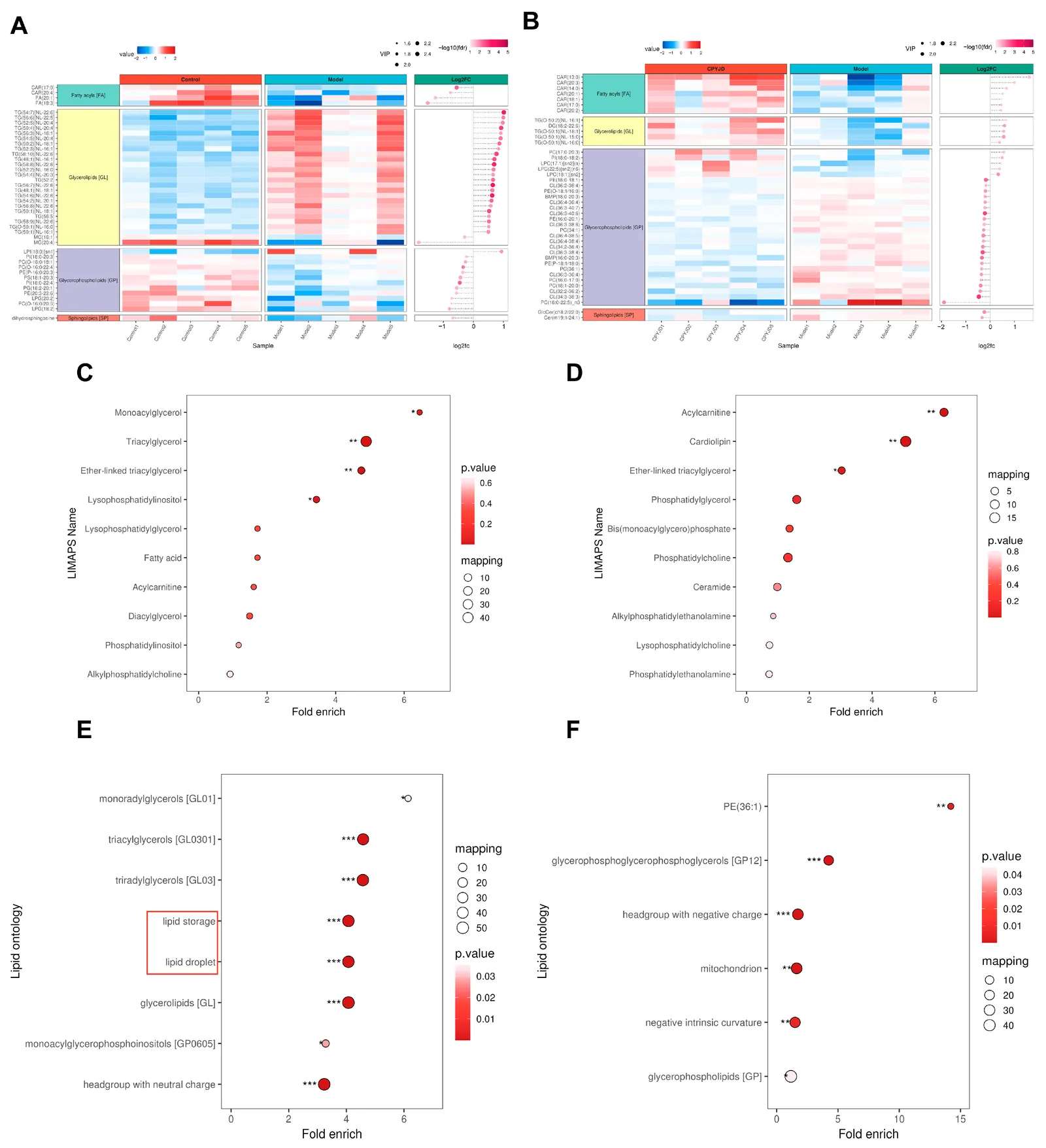
Effects of CPYJD on functional enrichment analysis of differentially expressed lipids. (**A**) Heatmap of significantly differential lipids between the control and model groups (*n* = 5 rats per group). (**B**) Heatmap of significantly differential lipids between the model and CPYJD-H groups (*n* = 5 rats per group). (**C**) Bubble plot of LIPID MAPS functional classification enrichment analysis between the control and model groups (*n* = 5 rats per group). (**D**) Bubble plot of LIPID MAPS functional classification enrichment analysis between the model and CPYJD-H groups (*n* = 5 rats per group). (**E**) Bubble plot of LION functional classification enrichment analysis between the control and model groups (*n* = 5 rats per group). The red square highlights the lipid storage and lipid droplet categories, which are most relevant to the focus of this study. (**F**) Bubble plot of LION functional classification enrichment analysis between the model and CPYJD-H groups (*n* = 5 rats per group). * *p* < 0.05, ** *p* < 0.01, *** *p* < 0.001.

**Figure 8:**
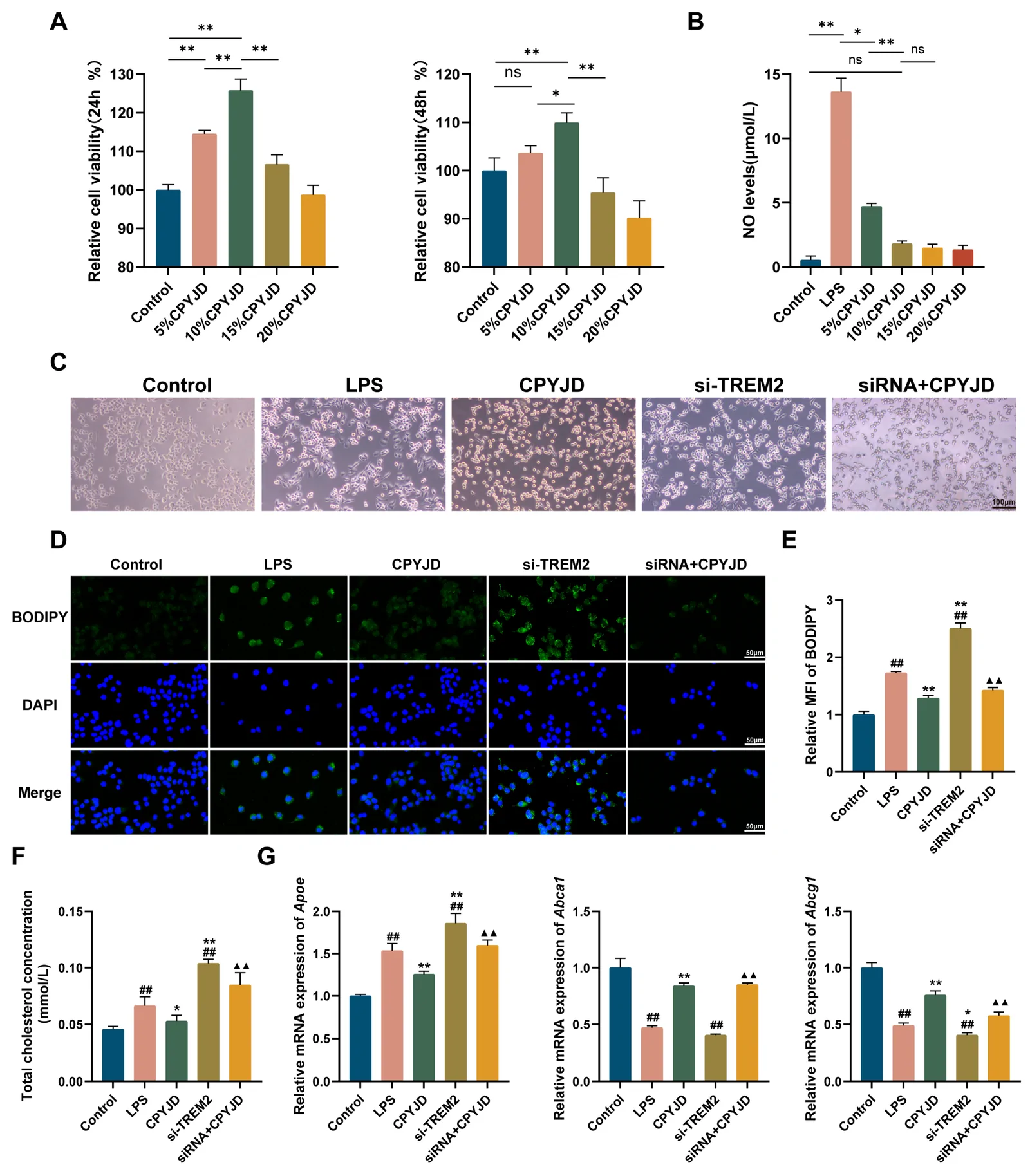
Screening of CPYJD-medicated serum concentration, morphological improvement of BV2 cells, and effects of CPYJD-medicated serum and TREM2 knockdown on lipid metabolism in LPS-stimulated BV2 cells. (**A**) Effects of CPYJD-medicated serum on BV2 cell viability at 24 and 48 h; cell viability was calculated relative to the concentration-matched blank serum (*n* = 3 independent experiments; LSD post-hoc test). (**B**) NO levels in the supernatant of BV2 cells measured by Griess assay (*n* = 3 independent experiments; Dunnett’s T3 post-hoc test). (**A**,**B**) ns, not significant; * *p* < 0.05, ** *p* < 0.01. (**C**) Morphology of BV2 cells observed by light microscopy (*n* = 3 independent experiments). Magnification × 100. (**D**) BODIPY signals in BV2 cells detected by fluorescent probe assay (*n* = 3 independent experiments). Magnification × 400. (**E**) Quantification of the MFI of BODIPY in BV2 cells (*n* = 3 independent experiments; LSD post-hoc test). (**F**) Total cholesterol concentration in BV2 cells (*n* = 3 independent experiments; LSD post-hoc test). (**G**) Relative mRNA expression of *Apoe*, *Abca1*, and *Abcg1* in BV2 cells (*n* = 3 independent experiments; LSD post-hoc test). Data are expressed as mean ± SD. (**E**–**G**) # *p* < 0.05, ## *p* < 0.01 vs. control group; * *p* < 0.05, ** *p* < 0.01 vs. LPS group; ▲ *p* < 0.05, ▲▲ *p* < 0.01 vs. si-TREM2 group.

**Figure 9:**
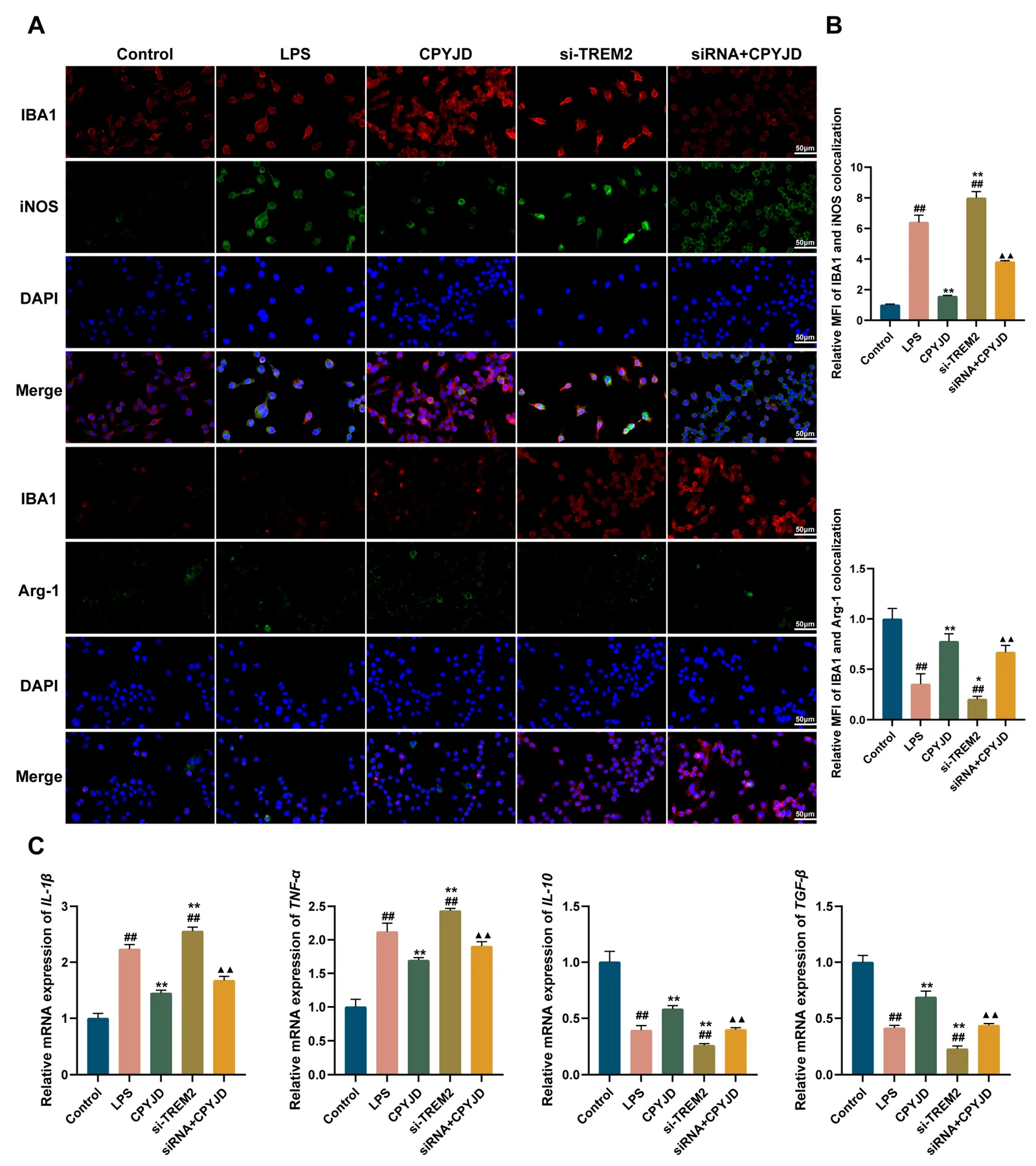
Effects of CPYJD-medicated serum and TREM2 knockdown on microglial phenotype and inflammatory responses in LPS-stimulated BV2 cells. (**A**) Immunofluorescence co-localization of IBA1 with iNOS and IBA1 with Arg-1 (*n* = 3 independent experiments). (**B**) Quantification of the MFI of IBA1/iNOS and IBA1/Arg-1 co-localization in BV2 cells (*n* = 3 independent experiments; LSD post-hoc test). (**C**) Relative mRNA expression of *IL-1β*, *TNF-α*, *IL-10*, and *TGF-β* in BV2 cells (*n* = 3 independent experiments; LSD post-hoc test). Magnification × 400. Data are expressed as mean ± SD. # *p* < 0.05, ## *p* < 0.01 vs. control group; * *p* < 0.05, ** *p* < 0.01 vs. LPS group; ▲ *p* < 0.05, ▲▲ *p* < 0.01 vs. si-TREM2 group.

**Figure 10:**
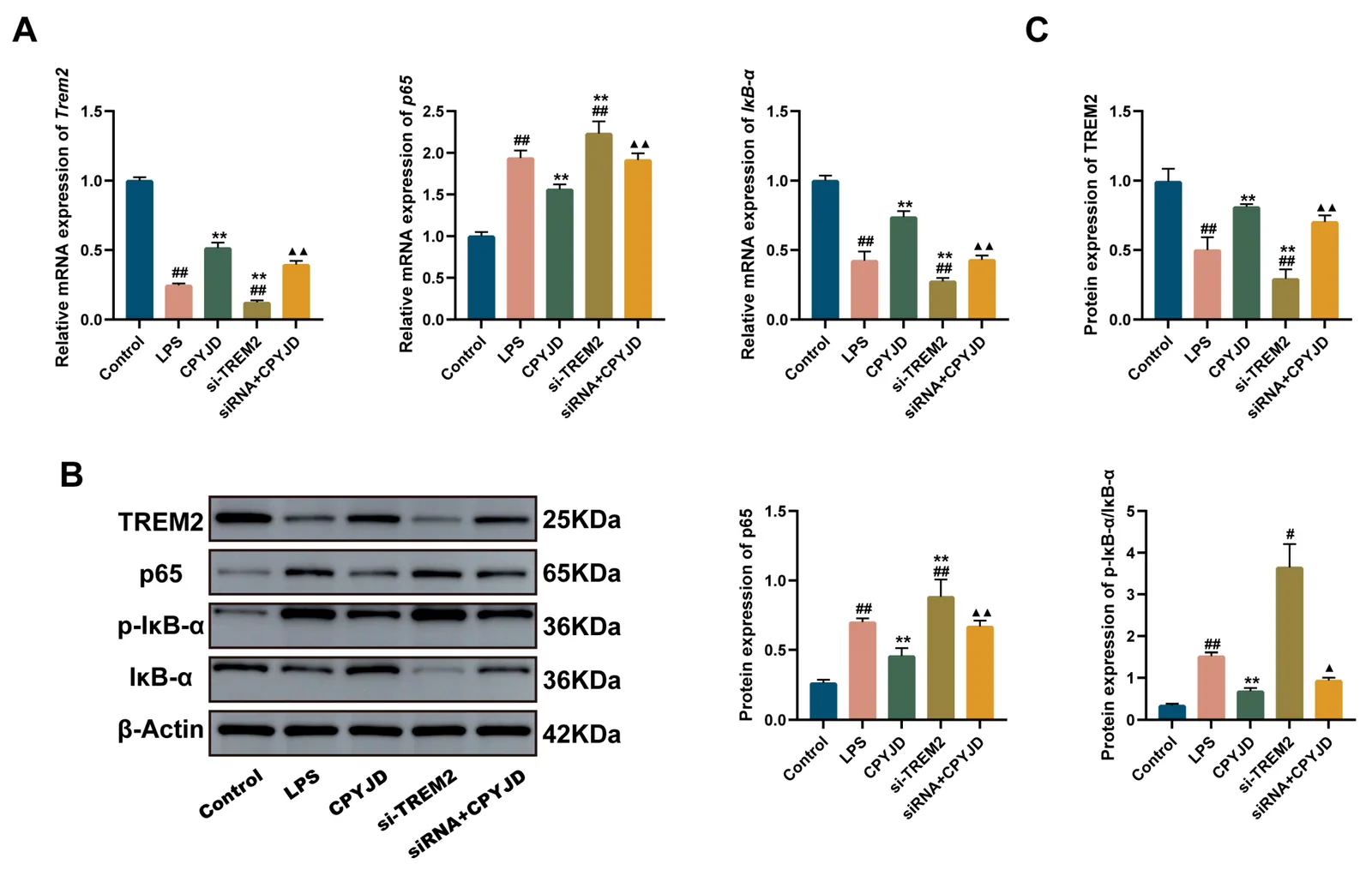
Effects of CPYJD-medicated serum on the expression of factors related to the TREM2/NF-κB signaling axis in BV2 cells. (**A**) Relative mRNA expression of *Trem2*, *p65*, and *IκB-α* in BV2 cells (*n* = 3 independent experiments; LSD post-hoc test). (**B**) Representative Western blot bands of TREM2, p65, IκB-α, and p-IκB-α (*n* = 3 independent experiments). (**C**) Quantification of protein expression of TREM2 and p65, and the p-IκB-α/IκB-α ratio in BV2 cells (*n* = 3 independent experiments; LSD, LSD, and Dunnett’s T3 post-hoc tests, respectively). Data are expressed as mean ± SD. # *p* < 0.05, ## *p* < 0.01 vs. control group; * *p* < 0.05, ** *p* < 0.01 vs. LPS group; ▲ *p* < 0.05, ▲▲ *p* < 0.01 vs. si-TREM2 group.

**Figure 11:**
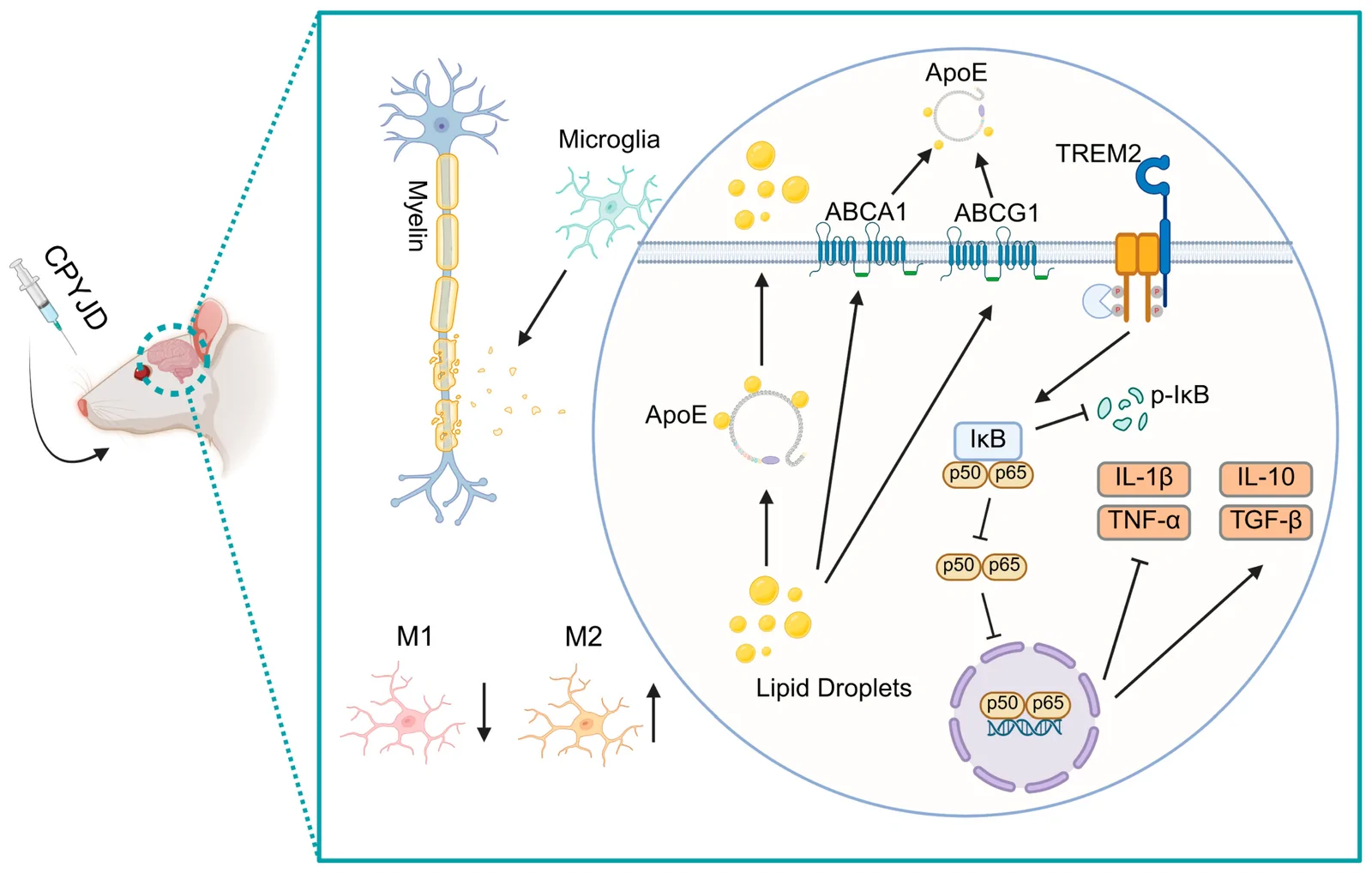
The possible mechanism of CPYJD in the treatment of TS. Arrows (→) indicate promotion or upregulation, and blunt-ended arrows (⊥) indicate inhibition. Created in BioRender. Yao, J. (2026) https://BioRender.com/hs9qcwd (accessed on 9 September 2026).

**Table 1:** Major compounds identified in CPYJD by UPLC-MS/MS. The parameters shown include compound name, molecular formula, retention time (RT), mass error, mass-to-charge ratio (*m*/*z*), ion mode, and relative peak area (%).

No.	Compound	Formula	RT (min)	Mass Error (ppm)	*m*/*z*	Ion Mode	Relative Peak Area (%)
1	Parishin E	C19H24O13	4.1548667	−1.15	459.113884	neg	9.90
2	Orobanone	C15H20O	9.6113000	0.40	217.158778	pos	8.14
3	Parishin C	C32H40O19	4.9511833	−0.56	727.208693	neg	5.80
4	Adenosine	C10H13N5O4	1.7655667	0.48	268.104158	pos	5.32
5	Arteannuin	C15H20O3	9.0844833	0.51	231.138084	pos	2.82
6	5-Hydroxymethylfurfural	C6H6O3	2.6489167	1.46	127.039155	pos	2.71
7	L--Glutamyl-S-[(4-hydroxyphenyl)methyl]	C17H23N3O7S	4.4840000	0.21	414.133034	pos	1.78
8	Curcumenol	C15H22O2	8.5465167	0.44	235.169358	pos	1.75
9	3′,6-Disinapoyl sucrose	C34H42O19	6.0889000	−0.43	753.224427	neg	1.74
10	6alpha-Hydroxytomentosin	C15H20O4	11.1320667	0.08	247.132892	pos	1.41
11	Isomaltotriose	C18H32O16	0.8426833	0.37	543.132377	pos	1.31
12	Angophorol	C18H18O5	6.9938000	−1.28	314.138823	pos	0.98
13	Adenine	C5H5N5	1.2148833	1.08	136.061918	pos	0.95
14	Tenuifolin	C36H56O12	7.8833667	−0.48	679.369571	neg	0.93
15	3,4-Dihydroxyphenylacetic acid	C8H8O4	2.8723167	−1.65	167.034705	neg	0.93
16	Polygalaxanthone XI	C25H28O15	5.4498833	0.56	569.150417	pos	0.89
17	Neoprocurcumenol	C15H22O2	8.2723667	0.32	235.169332	pos	0.89
18	13-Hydroxygermacrone	C15H22O2	9.1735667	0.16	235.169294	pos	0.88
19	Carabrolactone A	C15H22O5	8.7102000	0.43	247.132992	pos	0.88
20	4-(2-Nitroethyl)phenyl primeveroside	C19H27NO12	1.6517833	3.72	466.133642	pos	0.84

**Table 2:** The motor behavior and stereotyped behavior scale.

Score	Motor Behavior	Stereotyped Behavior
0	Quiet or normal activity	No stereotyped behavior
1	Overexcited	Body rotation (clockwise or counterclockwise)
2	Increased exploratory behavior with discontinuous sniffing	Excessive up or down movement of the head and neck (an abnormal movement of the head and neck in the vertical direction to the ground)
3	Nonstop running	Excessive up or down movement of the head and neck plus rotation
4	Nonstop running accompanied by fearful jumping	Side-to-side head weaving, accompanied by excessive up or down movement of the head and neck

**Table 3:** Primer information.

Organism	Primers	Up/Down Stream	Primer Sequence	Length (bp)
Rats	*Apoe*	F	TGACGGTACTGATGGAGGACACT	201
		R	CCAGCATGGTGTTTACCTCGTT	
	*Abca1*	F	CATCTCCCGACCCAGCATCT	116
		R	ATCACCTCCTGCCGCATGTC	
	*Abcg1*	F	CAACGTGGATGAGCTTGAGACA	182
		R	GGGTCTTGTATCCTTTCTTCCTCC	
	CD86	F	GTGTTTGAAGATGCAGAACCAGC	113
		R	GCAATTGGGGTTCAACTTCCTC	
	CD206	F	CATCTGCCAGCGACATAATAGC	196
		R	GGACACCAGGTTTCCTTTCAATC	
	*Trem2*	F	CCAAGCCCTCAACACCACA	201
		R	ACCGTGCTCCCATTCTGCTT	
	*p65*	F	AACGCAAAAGGACCTACGAGA	156
		R	TTGAAAAGGCATAGGGCTGG	
	*IκB-α*	F	CCCAAGTACCCGGATACAGCA	133
		R	GTCATCGTAGGGCAACTCATCTT	
	*GAPDH*	F	CTGGAGAAACCTGCCAAGTATG	138
		R	GGTGGAAGAATGGGAGTTGCT	
Mouse	*Apoe*	F	CAGATCAGCTCGAGTGGCAA	151
		R	TCCATCAGTGCCGTCAGTTC	
	*Abca1*	F	CGTGTCTTGTCTGAAAAAGGAGG	82
		R	CGTGTCACTTTCATGGTCGC	
	*Abcg1*	F	ACACCGATGTGAACCCGTTT	161
		R	CAGATGTGTCAGGACCGAGT	
	*IL-1β*	F	ATGCCACCTTTTGACAGTGATG	139
		R	TGATGTGCTGCTGCGAGATT	
	*TNF-α*	F	GATCGGTCCCCAAAGGGATG	92
		R	CCACTTGGTGGTTTGTGAGTG	
	*IL-10*	F	GCATGGCCCAGAAATCAAGG	162
		R	ACACCTTGGTCTTGGAGCTTATTA	
	*TGF-β*	F	ACTGGAGTTGTACGGCAGTG	123
		R	GGGGCTGATCCCGTTGATT	
	*Trem2*	F	CTGGAACCGTCACCATCACTC	183
		R	CGAAACTCGATGACTCCTCGG	
	*p65*	F	ACACCCCACCATCAAGATCA	176
		R	CTCTATAGGAACTATGGATACTGCG	
	*IκB-α*	F	GAGCTCCGAGACTTTCGAGG	149
		R	AGACACGTGTGGCCATTGTA	
	*GAPDH*	F	GGTTGTCTCCTGCGACTTCA	183
		R	TGGTCCAGGGTTTCTTACTCC	

## Data Availability

The original contributions presented in this study are included in the article. Further inquiries can be directed to the corresponding authors.

## Abbreviations

The following abbreviations are used in this manuscript:

ABCA1	ATP-binding cassette subfamily A member 1
ABCG1	ATP-binding cassette subfamily G member 1
ADHD	Attention-deficit/hyperactivity disorder
Arg-1	Arginase-1
ApoE	Apolipoprotein E
CAM	Complementary and alternative medicine
CBIT	Comprehensive Behavioral Intervention for Tics
CD86	Cluster of differentiation 86
CD206	Cluster of differentiation 206
CNS	Central nervous system
CPYJD	Changpu Yujin Decoction
CPYJD-L	Low-dose CPYJD
CPYJD-M	Medium-dose CPYJD
CPYJD-H	High-dose CPYJD
CSTC	Cortico-striatal-thalamic-cortical
DAM	Disease-associated microglia
ELISA	Enzyme-linked immunosorbent assay
GL	Glycerolipids
GP	Glycerophospholipids
HE	Hematoxylin–eosin
HRT	Habit reversal training
IBA1	Ionized calcium-binding adapter molecule 1
IDPN	3,3′-iminodipropionitrile
IF	Immunofluorescence
IHC	Immunohistochemistry
IL-1β	Interleukin-1β
IL-10	Interleukin-10
IκB-α	Inhibitor of nuclear factor kappa-B alpha
iNOS	Inducible nitric oxide synthase
LPS	Lipopolysaccharide
MFI	Mean fluorescence intensity
MG	Microglia
NDD	Neurodevelopmental disorder
NF-κB	Nuclear factor kappa-B
NO	Nitric oxide
NLRP3	NOD-, LRR- and pyrin domain-containing protein 3
OCD	Obsessive-compulsive disorder
p65	Transcription factor p65
PCA	Principal component analysis
PINK1	PTEN-induced kinase 1
QC	Quality control
ROS	Reactive oxygen species
RT-qPCR	Reverse transcription quantitative PCR
SD	Sprague-Dawley
SPF	Specific pathogen-free
TEM	Transmission electron microscopy
TGF-β	Transforming growth factor-β
TNF-α	Tumor necrosis factor-α
TREM2	Triggering receptor expressed on myeloid cells 2
TS	Tourette syndrome
UPLC-MS/MS	Ultra-performance liquid chromatography–tandem mass spectrometry
VIP	Variable importance in projection
WB	Western blot
YGTSS	Yale Global Tic Severity Scale
